# Chemsex and uptake of HIV pre-exposure prophylaxis among adult cisgender gay, bisexual and other men who have sex with men worldwide: a systematic review with meta-analysis

**DOI:** 10.1016/j.eclinm.2026.103804

**Published:** 2026-03-05

**Authors:** Joan Gil Miñana, Sonia Arias García, Elisa De Lazzari, Francisco José Montoya Conesa, Lorena De La Mora, Montserrat Laguno, Josep Mallolas, Maria Martínez Rebollar, Alexandra Calmy, Lucía González Fernández

**Affiliations:** a Faculty of Medicine, Institute of Global Health, University of Geneva, Chemin des Mines 9, 1202, Geneva, Switzerland; b The Joint United Nations Program on HIV/AIDS (UNAIDS), Av. Appia 20, 1211, Geneva, Switzerland; c HIV Unit, Infectious Diseases Service, Hospital Clinic-IDIBAPS, University of Barcelona, Carrer de Villarroel 170, 08036, Barcelona, Spain; d Centro de Investigación Biomédica en Red de Enfermedades Infecciosas (CIBERINFEC), Instituto de Salud Carlos III, Madrid, Spain; e HIV Unit, Infectious Disease Division, Department of Medicine, Geneva University Hospitals, Rue Gabrielle-Perret Gentil 6, 1205, Geneva, Switzerland; f Department of Migrant Health, Global Tuberculosis Program. Baylor College of Medicine, 1102 Bates Avenue, FC 630, Houston, TX, 77030, USA

**Keywords:** Gay, Bisexual and other men who have sex with men, Chemsex, HIV pre-exposure prophylaxis, HIV prevention, Harm reduction

## Abstract

**Background:**

There is a lack of summarized information regarding the uptake of pre-exposure prophylaxis (PrEP) among HIV negative gay, bisexual and other men who have sex with men (GBMSM) who engage in chemsex. This systematic review and meta-analysis aimed to summarize, assess the quality of existing evidence, estimate the prevalence of PrEP uptake among GBMSM who engage in chemsex, based on available global evidence; and evaluate whether engagement in chemsex is associated with increased odds of PrEP uptake in this population.

**Methods:**

We searched Medline, Embase, Cochrane Reviews and CENTRAL, APA PsycInfo, Scopus, and LILACS. We included studies reporting chemsex practices among adult cisgender GBMSM and PrEP uptake until July 1, 2025. Evidence quality was assessed using the Newcastle–Ottawa Scale tool. Chemsex exposure was analyzed using two timeframe groups: recent (past 6 months) and ever (any longer or unspecified timeframe). Prevalence and associations -odds ratio (OR)- were pooled in meta-analyses using a random-effect model. Inconsistency, sensitivity, and publication bias analyses were conducted. PROSPERO registration: CRD42024573871.

**Findings:**

Among 3988 records screened, 28 studies comprising 36,339 participants across three world regions were included: Western and Central Europe and North America, Asia and the Pacific, and Latin America; all were rated low to moderate quality. The overall prevalence of PrEP use was 39% (95% CI: 29–49%) among GBMSM who engaged in chemsex, with no significant differences between *recent* and *ever* chemsex timeframe groups. Sensitivity analyses and meta-regressions showed no significant effect of study publication year, country income group or world region. Overall chemsex was associated with higher odds of PrEP use (OR = 3.44, 95% CI: 2.70–4.38), with a significantly stronger association for *ever* chemsex (OR = 4.74, 95% CI: 3.48–6.46) than for *recent* chemsex (OR = 2.84, 95% CI: 2.11–3.81p < 0.05). Meta-regressions indicated significantly lower odds of PrEP use in studies from low- and middle-income countries for both *recent* (OR = 0.54, 95% CI: 0.31–0.94; p = 0.031) and *ever* (OR = 0.46, 95% CI: 0.21–0.99; p = 0.046) chemsex timeframes. Heterogeneity remained moderate to high across all analyses.

**Interpretation:**

Our findings suggest that PrEP use is relatively common among GBMSM who engage in chemsex globally and chemsex is consistently and strongly associated with increased PrEP uptake in this population. The association between chemsex and PrEP use was significantly higher in studies from high-income settings, where chemsex practices are better described and PrEP is more widely available for this group. The lack of standardized definitions in the field contributes to the high heterogeneity and strongly influences the generation of global evidence. This first systematic review and metanalysis in the field highlights the need to expand and integrate chemsex harm reduction and PrEP services for GBMSM, especially in low- and middle-income settings, to improve health outcomes and advance towards the global HIV elimination goals.

**Funding:**

Publication fees were sponsored by the Research Group of the HIV Unit, Infectious Disease Division, Department of Medicine, Geneva University Hospitals, Geneva, Switzerland. The Covidence membership and the fees for an academic librarian developing a professional systematic search strategy were sponsored by the Research Group of the HIV Unit, Infectious Diseases Service, Hospital Clinic, and IDIBAPS, University of Barcelona, Barcelona, Spain. No further funding was received for this study.

**Research in context**

**Evidence before this study**

Adult cisgender gay, bisexual, and other men who have sex with men (GBMSM) remain disproportionately affected by HIV globally and the use of HIV pre-exposure prophylaxis (PrEP) is recommended as a highly effective prevention strategy for this population. The intentional use of drugs to enhance sexual experiences in this group, referred as chemsex, is associated with increased sexual risk behaviors and HIV infection. Chemsex typically involves drugs such as methamphetamine, γ-hydroxybutyrate/γ-butyrolactone (GHB/GBL), or mephedrone.

Before this study, there was no systematic review of the literature or meta-analysis to synthesize the published evidence and examine the association of PrEP use among adult cisgender GBMSM who engage in chemsex. We searched Medline, Embase, Cochrane Reviews and CENTRAL, APA PsycInfo, Scopus, and LILACS from inception to Dec 4, 2024, and updated on July 1, 2025, without language or time restrictions. Searches combined indexing terms and free text for “men who have sex with men”, “chemsex”, and “pre-exposure prophylaxis”. Eligible studies reported prevalence or correlates of PrEP use among adult cisgender GBMSM engaging in chemsex.

**Added value of this study**

This is the first systematic review and meta-analysis to provide a global perspective on the relationship between chemsex and PrEP use among adult cisgender GBMSM. Across 28 studies from three world regions: Western and Central Europe and North America (WCENA); Asia and the Pacific (AP); and Latin America (LA), pooled estimates indicate more than threefold higher odds of PrEP use among this group. The association is stronger in the subgroup of adult cisgender GBMSM who reported engaging in chemsex at some point in their lifetime than among those who reported recent chemsex. Adult cisgender GBMSM who engaged in chemsex had lower odds of PrEP use in low- and middle-income countries (LMICs) compared with high-income settings. Chemsex practices may serve as a behavioral marker to identify individuals at higher risk of HIV infection and to support the incorporation of PrEP into HIV prevention services for this population.

**Implications of all the available evidence**

Evidence emanating from this study shows that engagement in chemsex among adult cisgender GBMSM is a global phenomenon and supports incorporating screening of sexualized substance use and harm reduction services into sexual health services for this population. Similarly, adult cisgender GBMSM who engage in chemsex have increased odds of using PrEP globally, however this intervention should be scaled up more widely to significantly decrease the risk of HIV infection in this group. Expanding culturally adapted HIV and harm reduction interventions related to chemsex for adult cisgender GBMSM would benefit from expanding existing evidence, especially in underrepresented regions including sub-Saharan Africa and Eastern Europe and Central Asia.

## Introduction

Human immunodeficiency virus (HIV) remains a significant global public health concern, posing an important health risk to key populations.^1^ According to the Joint United Nations Programme on HIV/AIDS (UNAIDS), gay, bisexual and other men who have sex with men (GBMSM) are particularly vulnerable to HIV and frequently experience human rights violations and lack adequate access to services.^2^ In 2022, GBMSM were up to 23 times more likely to acquire HIV compared to the general adult population (15–49 years of age), with a reported global median HIV prevalence of 8% and rates reaching 11% in eastern and southern Africa. In the same year, GBMSM accounted for an estimated 20% of new infections globally, reaching up to 42% in Asia and the Pacific region and 45% in Latin America.^3^^,^^4^ Among the factors linked to HIV vulnerability in adult cisgender GBMSM, recent evidence has identified chemsex as a key contributor.^5^

Despite the lack of a uniform definition, chemsex is a complex and culturally diverse global phenomenon^6^ that typically involves the intentional use of specific drugs, such as methamphetamine, mephedrone, γ-hydroxybutyrate/γ-butyrolactone (GHB/GBL), and, to a lesser extent, ketamine or cocaine, by GBMSM, to enhance or facilitate sexual experiences.^7^ Chemsex can be characterized as a specific subset of sexualized drug use (SDU), distinguished by its intentionality for sex and prolonged duration of sex. This phenomenon has been primarily described within, and disproportionately affects, GBMSM community.^8^ Chemsex is associated with high-risk sexual behaviors such as condomless sex, multiple partners, group sex, transactional sex, and polydrug use. When these practices occur in a poorly managed or unregulated context, they can lead to severe and multidimensional consequences including increased risk of HIV, hepatitis C and other sexually transmitted infections, as well as mental, physical, social and substance use-related health conditions,^9^^,^^10^ highlighting the urgent need for integrated and comprehensive health strategies to address chemsex-related risks.^11^ Understanding the motivations behind chemsex is equally crucial to fully grasp the phenomenon and guide effective interventions. Reported reasons range from those perceived as positive, such as seeking pleasure and enhanced sexual experiences, to others linked to structural and psychosocial challenges, including HIV-related stigma, internalized homophobia, serophobia, and coping with emotional distress. These latter factors are more strongly associated with problematic patterns and adverse outcomes, emphasizing the need for approaches that address both behavioral and psychosocial dimensions.^12^, ^13^, ^14^ Despite its significant health implications, the timing, onset, and patterns of chemsex engagement across settings remain poorly defined, limiting the knowledge in the field, development of targeted interventions and evidence generation.^15^

Given the heightened risk of HIV acquisition in adult cisgender GBMSM, this group is critical target for the scale-up of prevention strategies, including HIV pre-exposure prophylaxis (PrEP). The use of PrEP has demonstrated high efficacy in reducing the risk of HIV transmission among GBMSM and is currently widely recommended as a core component of HIV prevention packages^16^ through oral and injectable options that offer flexibility to accommodate diverse needs and lifestyles. However, global scale up remains limited,^17^ with a recent global meta-analysis estimating that only 11–16% of GBMSM had used PrEP according to available data,^18^ and the 2025 UNAIDS *Global AIDS Update* likewise noting that PrEP coverage remains far below international targets.^19^

With the global expansion of HIV prevention strategies, including PrEP,^20^ targeting adult cisgender GBMSM, and the increasing use of chemsex,^21^ a growing body of literature explores the intersection between chemsex engagement and PrEP uptake in this group. Because individuals who engage in chemsex frequently meet behavioral eligibility criteria for PrEP, an association between chemsex and PrEP use is plausible. However, to date, the field lacks a summary of the published evidence that offers a global perspective. Therefore, this systematic review and meta-analysis aim to synthesize available evidence on PrEP use among adult cisgender GBMSM who engage in chemsex, estimating the prevalence of PrEP use and assessing whether chemsex engagement is associated with higher odds of PrEP use compared to those who do not engage in chemsex.

## Methods

### Study design

This systematic review with meta-analysis followed the guidelines provided by the Preferred Reporting Items for Systematic Reviews and Meta-Analysis Protocols (2020 PRISMA-P).^22^ The review was registered in the International Prospective Register of Systematic Reviews (PROSPERO) with the registration ID CRD42024573871.

### Search strategy

The search strategy was co-designed with the assistance of a scientific information specialist librarian from the University of Zurich medical library. The electronic databases Medline (via Ovid), Embase (via Elsevier), Cochrane Reviews and CENTRAL (via Cochrane Library/Wiley), APA PsycInfo (via EBSCOhost), Scopus (via Elsevier), and LILACS (via VHL Regional Portal) were searched using a combination of medical indexing terms and free text terms for the concepts “men who have sex with men”, “chemsex”, and “pre-exposure prophylaxis” including related phrases and synonyms. For the concept “pre-exposure prophylaxis”, specific therapy names were tested but ultimately not included in the search strategy, to avoid bias. The concepts included in the PICO question and full search strategy are reported in eTable 1 and eTable 2. Literature search was initially performed on December 4th, 2024, and updated on July 1st, 2025, except for LILACS which was not accessible on that date. Search results from each database were exported, uploaded individually, and deduplicated using Covidence systematic review software (Veritas Health Innovation, Melbourne, Australia).^23^ No dates, geography, or language restrictions were applied during the search or selection process. Additionally, backward and forward reference search was conducted.

### Study selection criteria

The studies selection criteria followed the Population, Intervention, Comparison, and Outcome (PICO) design (eTable 1). We included cross-sectional studies involving HIV-negative adult cisgender GBMSM reporting at least once or current chemsex practices (self-reported) and PrEP use. Our comparator encompassed adult cisgender GBMSM who reported no engagement in chemsex practices and use PrEP. Only studies reporting quantitative data were included; studies without quantitative outcomes relevant to the review were excluded at full-text screening. Reviews, case reports, randomized controlled trials, and editorials were excluded. Gray literature sources, such as preprints or conference abstracts, were also excluded. Before formal screening, a pilot calibration exercise was conducted in which all reviewers independently screened a sample of studies to test, redefine, and align on the eligibility criteria. Two independent reviewers screened titles and abstracts, and performed full text review (JGM, SAG). Discrepancies were solved through discussion involving a third reviewer (JGM, SAG, LGF).

### Definition of exposure and outcome

The studies included different definitions for chemsex practices (exposure) and PrEP use (outcome). The definitions of chemsex have evolved over time and vary considerably across studies, reflecting differences in drug use patterns, sexual contexts, and regional terminology. The growing role of digital platforms in facilitating encounters has also contributed to the evolving nature of chemsex.^24^ Broadly, chemsex refers to the intentional use of specific non-prescription substances, most commonly methamphetamine, GHB/GBL, and mephedrone, to initiate, enhance, or prolong sexual encounters. However, the scope of the term expanded in recent years to include a wider range of substances and behaviors. In different settings, various terms are used to describe similar practices, for instance, *party and play (PnP)* in the United States, *ice parties* or *high fun* in Southeast Asia, *chills* in Spain or *festinha* in Brazil and *fiesta química* in some parts of Latin America and the Caribbean. Given the lack of a standardized definition across the literature, for this review, we included studies that used different definitions of *chemsex*, provided that the core components were present: intentional drug use of *typical chemsex drugs* for the purpose to enhance sexual encounters among GBMSM and clearly were differentiated from other patterns of substance use. As such, we included studies that *explicitly* described chemsex practices and studies that referred to chemsex *implicitly,* where the term chemsex was not alluded to, however, they reported the use of drugs typically associated within sexual contexts. Regarding the timeframe of chemsex, participants self-reported their last engagement over heterogeneous periods of time, ranging from the previous 3 months to at some point in their lifetime, or without a specified period. For the purpose of this review, chemsex engagement was classified as either *recent chemsex* (reported within the past 6 months) or *ever engaged in* chemsex (hereafter referred to as “ever chemsex”), which included all other reported periods such as 12 months, 18 months, lifetime, or unspecified timing. This latter category may also include individuals who engaged in chemsex more recently, although this could not be distinguished based on the available data. PrEP use was typically described as the use of oral Tenofovir-based PrEP or long-acting injectable PrEP (cabotegravir). The studies differed in how PrEP use was reported, including daily oral, on-demand, injectable, or even informal use. For the purpose of this review, PrEP uptake was recorded when the participants self-reported use of any modality of PrEP. Continuation, adherence, or re-initiation of PrEP was not analyzed.

### Data extraction

A standardized data extraction form was used to collect information from the included studies. Study data were collected and managed using REDCap electronic data capture tools hosted at Hospital Clínic Barcelona.^25^ One reviewer (SAG) conducted the data extraction, and a second reviewer (JGM) cross-verified all extracted data. None of the included studies required translation. We collected the following information: first author, publication year and journal, study design, country, setting, study population, study period, definition and timeframe for chemsex exposure, definition of PrEP and timeframe of use when available, and study primary endpoint. We extracted the number of participants that reported using chemsex and PrEP, and those who did not report using chemsex or PrEP when participants answered both questions. Where available, we extracted unadjusted effect measures using odds ratios (OR), along with their 95% confidence intervals. If OR was not reported, we used extracted data and calculated the unadjusted OR. Additionally, we documented covariates included in adjustments. For studies that presented only p-values without corresponding OR, we computed unadjusted effect measures when sufficient data were available. If key data were missing or unclear, study authors were contacted. In cases where no response was received, the limitation was documented, and the study was excluded from the quantitative synthesis.

### Quality assessment of studies

We assessed study quality using the Newcastle–Ottawa Scale (NOS) quality assessment tool adapted for observational, cross-sectional studies.^26^ Studies were evaluated based on three broad criteria: selection of study groups (up to 5 points), comparability of groups (up to 1 point) and ascertainment of exposure or outcomes (up to 3 points) with a maximum possible score of 9 (eTable 3). Two reviewers independently evaluated each study, and discrepancies were resolved with a third reviewer (JGM, SAG, LGF). Based on the NOS point system, we assigned an overall quality rating as low = 0–3, moderate = 4–6 or high = 7–9.

### Statistical analysis and meta-analyses

A meta-analysis of prevalence estimates of PrEP use among individuals engaged in chemsex was first conducted using a logistic-normal random-effects model to account for between-study heterogeneity. The Freeman-Tukey double arcsine transformation was applied to stabilize variance, and pooled prevalence estimates with 95% Clopper-Pearson exact confidence intervals were back-transformed for interpretation. Statistical heterogeneity was tested using the I^2^ statistic, which describes the percentage of total variation in the effect sizes across studies due to heterogeneity rather than chance. I^2^ values up to 25% indicate no heterogeneity; 25%–50%, low; 50%–75%, moderate and 75%–100%, high heterogeneity.^27^ Potential publication bias in the prevalence meta-analysis was evaluated using a DOI plot and the Luis Furuya-Kanamori (LFK) index.^28^ The DOI plot provides a visual assessment of asymmetry, while the LFK index quantifies the degree of asymmetry: an LFK index < ±1 indicates no asymmetry and suggests no evidence of publication bias; values between ±1 and ±2 reflect minor asymmetry, indicating possible minor publication bias; values greater than ±2 denote major asymmetry, suggesting substantial publication bias may be present. Subsequently, a meta-analysis of ORs for PrEP use among individuals engaged in chemsex versus those not engaged was performed using a random-effects model based on restricted maximum likelihood. Results were displayed in a forest plot. Publication bias was assessed using a funnel plot and Harbord's test for small-study effects. To explore potential sources of heterogeneity, meta-regression analysis was conducted using selected study-level covariates: study publication year, country income group, world region and timeframe of chemsex exposure (*recent* versus *ever*), as moderators.

Subgroup analyses were also performed by timeframe of chemsex exposure to compare prevalence and associations across these groups. The study year refers to the year of publication. The world region where the study was undertaken was defined following UNAIDS classification^29^: Asia and the Pacific, eastern and southern Africa, eastern Europe and central Asia, Latin America and the Caribbean, Middle East and North Africa, western and central Africa and, western and central Europe and North America. Country income groups were defined according to the World Bank classification^30^ as high-income countries (HICs) and low- and middle-income countries (LMICs). The model included both within- and between-study variability, and heterogeneity explained was assessed using the adjusted R^2^. A test of residual heterogeneity was also performed. Additionally, a leave-one-out sensitivity analysis was conducted to identify influential studies. All tests were two-tailed, and statistical significance was set at p < 0.05. Statistical analyses were performed using Stata version 19.^31^

### Ethics statement

This study is a systematic review and meta-analysis of previously published, anonymized data. As no primary data were collected and no individual-level identifiable information was accessed, ethics approval was not required.

### Informed consent

Informed consent was not required for this study because it involved secondary analysis of data from published studies only.

### Role of the funding source

This study received financial support for publication fees from the Research Group of the HIV Unit, Infectious Diseases Division, Department of Medicine, Geneva University Hospitals, Geneva, Switzerland. The Covidence membership and the fees for the academic librarian who developed the systematic search strategy were sponsored by the Research Group of the HIV Unit, Infectious Diseases Service, Hospital Clínic, and IDIBAPS, University of Barcelona, Barcelona, Spain. No additional funding was received for this study. The funders of the study had no role in study design, data collection, data analysis, data interpretation, or writing of the report.

## Results

**Fig. 1:**
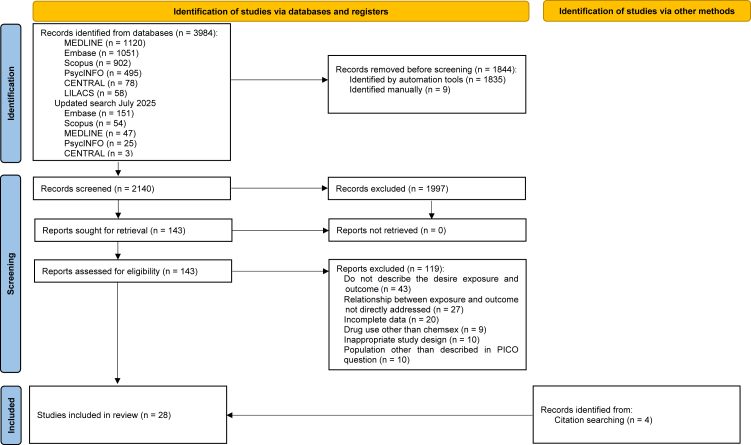
**PRISMA 2020 flow diagram for new systematic reviews which included searches of databases, registers and other sources**.

The search strategy identified 3984 records from electronic databases. Following the removal of 1844 duplicates, a total of 2140 records were screened by titles and abstracts. Of these, 1997 were excluded. The remaining 143 full-text articles were reviewed, of which 119 did not meet the eligibility criteria. A total of 42 authors were contacted; 12 provided the requested data, of which 10 met the inclusion criteria. An additional 4 articles were identified via citation searching. A total of 28 studies were finally included in this review, as outlined in the PRISMA flow diagram (Fig. 1). Reasons for exclusion are presented in eTable 4.

### Characteristics of included studies, participants and quality of studies

Table 1 provides a summary of the 28 included studies, all (100%)^32^, ^33^, ^34^, ^35^, ^36^, ^37^, ^38^, ^39^, ^40^, ^41^, ^42^, ^43^, ^44^, ^45^, ^46^, ^47^, ^48^, ^49^, ^50^, ^51^, ^52^, ^53^, ^54^, ^55^, ^56^, ^57^, ^58^, ^59^ were observational and cross-sectional, in the form of surveys. 14 studies (50%)^32^^,^^33^^,^^35^^,^^36^^,^^39^, ^40^, ^41^, ^42^, ^43^^,^^45^^,^^49^^,^^53^^,^^54^^,^^58^ were conducted in Western and Central Europe and North America (WCENA): Spain (n = 4)^33^^,^^45^^,^^53^^,^^54^, Netherlands (n = 3)^32^^,^^40^^,^^43^, United Kingdom (n = 3),^35^^,^^41^^,^^49^ France (n = 1),^42^ Italy (n = 1),^58^ Portugal (n = 1),^39^ and United States (n = 1).^36^ 12 studies (43%)^25^^,^^28^^,^^35^^,^^38^^,^^39^^,^^41^, ^42^, ^43^^,^^46^, ^47^, ^48^^,^^50^ were held in Asia and the Pacific (AP): China, Hongkong,^37^^,^^59^ Mainland,^55^ and Taiwan^57^ (n = 4), Australia (n = 2),^34^^,^^47^ Malaysia (n = 2),^44^^,^^48^ India (n = 1),^51^ New Zealand (n = 1),^52^ Pakistan (n = 1),^50^ and Thailand (n = 1).^56^ Only two studies (7%) occurred in Latin America (LA), both in Brazil (n = 2).^38^^,^^46^ Of the 28 included studies, 20 (71%) were conducted in high-income settings,^32^, ^33^, ^34^, ^35^, ^36^, ^37^^,^^39^, ^40^, ^41^, ^42^, ^43^^,^^45^^,^^47^^,^^49^^,^^52^, ^53^, ^54^^,^^57^, ^58^, ^59^ and 8 (29%) in low- and middle-income settings.^38^^,^^44^^,^^46^^,^^48^^,^^50^^,^^51^^,^^55^^,^^56^

**Table 1:** Characteristics of the included studies.

Study		Country	Study setting and participants	Study primary endpoint	Study period	Exposure engagement in chemsex			Outcome^a^ PrEP uptake		Sample size
First author	Publication year					Definition	Drug reported	Time frame	Definition in study	Time frame	
Coyer L.	2018	Netherlands	Prospective cohort study. HIV-negative MSM participating in Amsterdam Cohort Studies	Trends in PrEP use, PrEP eligibility, and intention to use PrEP	2015–2017	Use of drugs during sex with casual partners	GHB/GBL, mephedrone, methamphetamine	6 months	Daily or event-driven PrEP (self-obtained or via studies)	6 months and lifetime PrEP use	687
Valencia J.	2018	Spain	Cross-sectional study. HIV-negative MSM who attended ‘Orgullo Gay Madrid 2016″	Prevalence of drug use and chemsex-related drug use, and associated factors, including PrEP	2016	Use of drugs associated with chemsex	GHB/GBL, mephedrone, methamphetamine	12 months	PrEP use history	Lifetime PrEP use	666
Hammoud MA.	2019	Australia	Prospective cohort study. MSM recruited online participating in the Flux study	Incidence of PrEP uptake, initiation predictors, and characteristics associated with non-uptake	2014–2017	Recent drug use to enhance sex	Methamphetamine	6 months	Daily, “every other day” or event-driven PrEP	Daily, every other day, or before and after sex	1257
Hanum N.	2020	UK	Prospective cohort study. HIV-negative or of unknown HIV status MSM participating in the AURAH2 study	PrEP and PEP awareness and use among HIV-negative MSM and predictors of PrEP initiation	2013–2018	Use of at least one chemsex-related drug	Crystal methamphetamine, γ-hydroxybutyrate (GHB), γ-butyro- lactone (GBL), or mephedrone	3 months	PrEP use history	12 months	1162
Okafor CN.	2020	United States	Observational subanalysis within a randomized clinical trial. Black MSM participating in the HPTN 073 randomized clinical trail	PrEP adherence and initiation in relation to substance use	2013–2014	Self-reported use of different drugs before/during CAS	Alcohol, marijuana, inhaled nitrates, cocaine, and methamphetamine	3 months	Tenofovir-based daily PrEP initiation or continuation	Not specifically reported	226
Wang Z.	2020	China (Hong Kong)	Cross-sectional study. MSM self-reported as HIV-negative or of unknown HIV status	PrEP uptake and willingness among sexualized drug users	April–December 2018	Sexualized drug use in the last year (types of drugs used, poly-drug use, time since first episode, CAS, frequency, use of drug rehabilitation service)	Ketamine, methamphetamine, cocaine, cannabis, ecstasy, dormicum, Halcion, Erimin 5, hypnotic drugs (non- prescription), heroin, cough suppressant, amyl nitrite GHB/GBL methoxy-N, N-diisopropyltryptamine (Foxy), mephedrone	12 months	Current use of PrEP, sources of PrEP and methods of PrEP use	Not specifically reported	580
Blair K.	2021	Brazil	Cross-sectional. PrEP-eligible MSM	Current PrEP uptake, and key predictors: HIV knowledge, and internalized homonegativity	February–March 2020	Illicit substance use before or during sex	Cocaine, crack, basic paste, or oxy; marijuana, hashish, or skank; ecstasy or MD, methamphetamines (crystal or speed); GHB or GBL; poppers; other inhalants; mephedrone; hallucinogens (LSD, mushroom tea)	6 months	Current use of PrEP	Not specifically reported	2398
Chone JS.	2021	Portugal	Prospective cohort study. MSM recruited online during COVID-19 pandemic	Prevalence and factors associated with chemsex, including PrEP use, during the COVID-19 social distancing	May 2020	Drug use immediately before and/or during sex	Alcohol, opioids, cannabis, sedatives, cocaine, stimulants, hallucinogens, and poppers	3 months	Tenofovir-based oral PrEP	Not specifically reported	1301
Hulstein SH.	2021	Netherlands	Cross-sectional study. MSM reporting informal PrEP use	Eligibility for PrEP, intention to use PrEP, informal use of PrEP, chemsex and STI	2017–2018	Self-reported use of at least one of the following substances in the context of sex	GHB, methamphetamine, and/or mephedrone	6 months	Informally obtained Tenofovir-based oral PrEP obtained via acquaintance, online, buyers' club, on travel, or other options. Used daily and event-driven	3 months	5120
Hyndman I.	2021	UK	Cross-sectional study. Online survey of HIV-negative MSM attending Dean Street clinic during COVID-19	Impact of COVID-19 on sexual behavior, and mental well-being. Variables compared by PrEP use	March–June 2020	Sexualized recreational drug use	Not specifically reported	3 months	Self-reported PrEP use during lockdown	Not specifically reported	814
Rollet D.	2021	France	Cross-sectional study. MSM attending 6 STI clinics in Paris.	Number of general practitioner visits reported by patients (compared chemsex users and controls). Evaluated PrEP follow-up.	October–December 2018	Drug use in a sexual context (excluding alcohol, poppers and cannabis)	Tobacco, cocaine, MDMA, crystal, methamphetamine, Cathinones, GHB/GBL, ketamine, opiates, hallucinogens	12 months	PrEP use history and PrEP follow-up	Not specifically reported	364
Coyer L.	2022	Netherlands	Prospective cohort study. HIV-negative MSM after PrEP-initiation with controls not initiating PrEP	Number of casual partners, proportion engaging in CAS and receptive CAS with casual partners, sexualized drug use, any STI, and anal STI	2015–2019	Use of drugs during sex	Mephedrone, methamphetamine, GHB/GBL, ketamine, amphetamine, cocaine, ecstasy/MDMA	6 months	PrEP initiation, including study contexts and outside the ACS, informal or prescribed PrEP. Used daily and event-driven	Not specifically reported	858
Eger WH.	2022	Malaysia	Cross-sectional study. MSM recruited online	PrEP use and associated factors influencing its uptake	June–July 2020	Drug use before sexual intercourse	Crystal, methamphetamine, ketamine, ecstasy, and gamma-hydroxybutyrate “G”	6 months	History of PrEP use, as “Yes” or “No”	Not specifically reported	355
García-Pérez JN.	2022	Spain	Cross-sectional study. MSM who attended a sexual health clinic in Barcelona	Illicit drug and chemsex use, sexual behavior, and STI prevalence; associations between drug use, PrEP use, and STI	January–June 2019	Any recreational drug use with a sexual purpose independently of the drug type or number of drugs used	Poppers, cannabis, MDMA, cocaine, GHB, amphetamines, ketamine, methamphetamine, mephedrone, LSD	12 months	PrEP use history	12 months	514
Jalil EM.	2022	Brazil	Cross-sectional study. HIV-negative MSM recruited online/on-site	Prevalence and predictors of sexualized drug use, including PrEP use, among gender minorities	October–December 2020	Any use of drug before or during sex	Tobacco, marijuana, cocaine, crack, ecstasy, amphetamines, ketamine, meth, GHB, poppers, other inhalants	6 months	History or current PrEP use. Daily, event-driven or injectable PrEP (cabotegravir).	Not specifically reported	3553
MacGibbon J.	2022	Australia	Cross-sectional study. HIV-negative MSM recruited online	Factors associated with PrEP use among men in relationships	April–June 2021	Any recreational drug use with a sexual purpose	MDMA, GHB, Crystal	6 months	Daily or event-driven current PrEP use	Not specifically reported	1185
Maviglia F.	2022	Malaysia	Cross-sectional study. MSM recruited online	Prevalence and associated factors with chemsex, including PrEP use	August–September 2021	Any use of drug before or during sex	Ecstasy, crystal, methamphetamine/ice, GHB/GBL, Foxy	6 months	PrEP knowledge and current, ever use history	Not specifically reported	870
Ogaz D.	2022	UK	Cross-sectional study. HIV-negative/unknown MSM recruited from London commercial venues	PrEP usage patterns and unmet needs	June–August 2019	Any use of drug before or during sex	Ketamine, GHB/GBL, mephedrone, methamphetamine	12 months	Self-reported PrEP use	12 months^b^	1408
Ali U.	2023	Pakistan	Cross-sectional study. HIV-negative MSM recruited online	Knowledge, willingness and history of PrEP use	September–November 2021	Sex with use of drugs	Amphetamine-type stimulants	6 months	Knowledge, willingness and history of PrEP use	Not specifically reported	347
Agarwal H.	2024	India	Cross-sectional study. Individuals who reported sex with men recruited via app	PrEP use and awareness	May–June 2022	Use of “party drugs”	Not specifically reported	Not specified	History or current PrEP use	Not specifically reported	3116
Andrews S.	2024	New Zealand	Cross-sectional study. MSM recruited online	Sexual practice, drug use and sociodemographic, social milieu and behavioral predictors of sexualized drug use	December 2018–February 2019	Illicit drug use motivated by ‘better sex’ and/or ‘party and play session’	Cannabis, alkyl nitrite,MDMA, methamphetamine, cocaine, LSD, GHB, amphetamine, other hallucinogen, ketamine, synthetic cannabis, heroin, mephedrone	6 months	Current use of PrEP	Not specifically reported	836
Íncera-Fernández D.	2024	Spain	Cross-sectional study. MSM recruited via networks and apps	Prevalence and association of sexualized drug use with sexual behaviors, prevention measures for HIV, and STI	Not specified	Intentional use of drugs for sexual purposes	Alcohol, cannabis, cocaine, poppers, ecstasy, erectile dysfunction medication, MDMA, GHB/GBL, meth, mephedrone, ketamine, heroin, benzodiazepines	18 months	History of PrEP use. Either prescribed or without prescription	18 months	493
Moreno-García S.	2024	Spain	Cross-sectional study. HIV-negative MSM recruited online.	Daily PrEP prevalence and associated factors	May–July 2020	Use of drugs within the 6 h prior to or during anal sex	Mephedrone, methamphetamine, GHB, ketamine	Lifetime	Current daily oral PrEP	Not specifically reported	4692
Sun J.	2024	China	Cross-sectional study. MSM recruited online via gay service groups.	Impact of SDU, particularly chemsex, on sexual behavior, HIV/STI infection risk, and the use PrEP among MSM	March–April 2022	Use of drugs for sexual purposes or in conjunction with sexual activities	Poppers, methamphetamine, ketamine, ecstasy, GHB/GBL	Lifetime	History of PrEP use (type not specified) and willingness to use PrEP.	Lifetime^b^	796
Boonruang J.	2025	Thailand	Longitudinal study. MSM in Bangkok, recruited from healthcare setting	Prevalence and patterns of amphetamine-type stimulant use among MSM, and associated factors (including PrEP use)	January 2018–May 2019	Sex with use of drugs	Amphetamine-type stimulants use	6 months	History and willingness to use PrEP	6 months	1375
Mayo D.	2025	China (Taiwan only)	Cross-sectional study. HIV-negative/unknown MSM recruited online	PrEP engagement and its predictors, including chemsex	April–May 2023	Use of drugs immediately before or during sex	Methamphetamine, poppers, GHB/GBL, ketamine, methcathinone/mephedrone	6 months	PrEP use and continuation	12 months	284
Pessina R.	2025	Italy	Cross-sectional study. MSM recruited online via LGBT + community settings and sexual health services in Italy.	Epidemiological profile of chemsex and related psychological and health factors	March–June 2023	Use of psychoactive substances, typically through oral or inhalation routes, during sexual activity	Mephedrone, GHB/GBL, methamphetamine	Lifetime	History of PrEP use	Not specifically reported	841
Wong N.	2025	China (Hong Kong only)	Cross-sectional study. PrEP-naïve and PrEP-experienced MSM recruited online through NGOs.	Interrelationship between chemsex engagement and PrEP use	February 2022–January 2024	Use of drugs with or without other sexualized drugs before or during sex	Methamphetamine and/or GHB	Lifetime	History of PrEP use	Lifetime or recent	338

HIV: human immunodeficiency virus; GBMSM: gay, bisexual and other men who have sex with men; GHB: gamma hydroxybutyrate; GBL: gamma butyrolactone; PrEP: Pre-exposure prophylaxis; PEP: post-exposure prophylaxis; CAS: condomless anal sex; STI: sexually transmitted infections; MDMA or MD: methylenedioxy-methylamphetamine. LSD: Lysergic Acid Diethylamide.

Transgender and non-binary people were excluded from this study, according to exclusion and inclusion criteria of the present study.

a All studies assessed oral PrEP use. One study (Jalil 2022) also mentioned long-acting injectable PrEP (cabotegravir).

b As reported by the author upon contact.

With regards to the chemsex practices, a total of 17 (61%) studies^32^^,^^33^^,^^35^^,^^39^, ^40^, ^41^, ^42^^,^^44^, ^45^, ^46^, ^47^, ^48^^,^^50^^,^^54^^,^^57^, ^58^, ^59^ explicitly described the use of chemsex; and 11 (39%) studies^34^^,^^36^, ^37^, ^38^^,^^43^^,^^49^^,^^51^, ^52^, ^53^^,^^55^^,^^56^ referred to it implicitly. Studies also captured the timeframe of chemsex: 17 (61%) studies reported *recent* chemsex^32^^,^^34^, ^35^, ^36^^,^^38^, ^39^, ^40^, ^41^^,^^43^^,^^44^^,^^46^, ^47^, ^48^^,^^50^^,^^52^^,^^56^^,^^57^^,^ among these: 4 (14%)^35^^,^^36^^,^^39^^,^^41^ and 13 (46%)^32^^,^^34^^,^^38^^,^^40^^,^^43^^,^^44^^,^^46^, ^47^, ^48^^,^^50^^,^^52^^,^^56^^,^^57^ included participants who engaged in chemsex in the previous 3 and 6 months, respectively. 11 studies reported *ever* chemsex timeframes^33^^,^^37^^,^^42^^,^^45^^,^^49^^,^^51^^,^^53^, ^54^, ^55^^,^^58^^,^^59^: 5 (18%)^33^^,^^37^^,^^42^^,^^45^^,^^49^ and one (4%)^53^ studies included participants who engaged in chemsex in the previous 12 and 18 months, respectively. Also, one (4%) study^51^ included participants who engaged in chemsex in a non-specified period, and 4 studies (14%)^54^^,^^55^^,^^58^^,^^59^ included participants that reported use of chemsex in their lifetime (unspecified). All studies (100%) reported oral Tenofovir-based PrEP use, and one (4%) study^46^ also referred to long-acting injectable PrEP (cabotegravir). None of the studies explicitly reported frequency, dosage, or patterns of chemsex use.

In total, 36,339 HIV-negative adult cisgender GBMSM were included across all the studies. The studies where participants reported recent chemsex contributed with 22,531 individuals while the studies where participants reported ever chemsex timeframes contributed with 13,808 individuals. A total of 5690 of the total 36,339 (16%) participants reported chemsex, and 5881 out of 36,339 (16%) participants reported the use of PrEP.

With regards to the quality of the evidence, of the 28 studies, 9 (32%) were rated as low quality,^36^^,^^38^^,^^41^^,^^44^^,^^50^, ^51^, ^52^^,^^57^^,^^59^ 19 (68%) as medium quality.^32^, ^33^, ^34^, ^35^^,^^37^^,^^39^^,^^40^^,^^42^^,^^43^^,^^45^, ^46^, ^47^, ^48^, ^49^^,^^53^, ^54^, ^55^, ^56^^,^^58^ Overall scores ranged from 1 to 6, with a median of 4.5. The most frequent methodological limitations were observed in the *Selection* domain, particularly regarding response rates, which were satisfactory in only 2 studies (7%)^33^^,^^56^ and, to a lesser extent, concerns about the representativeness of cases, which was adequate in 13 studies (46%).^32^^,^^33^^,^^35^, ^36^, ^37^^,^^40^^,^^42^^,^^43^^,^^45^^,^^46^^,^^49^^,^^55^^,^^56^ In the *Comparability* domain, only 10 studies (36%)^37^^,^^39^^,^^42^^,^^45^^,^^46^^,^^48^^,^^52^^,^^53^^,^^55^^,^^59^ adequately controlled potential confounders. In the *Outcome* domain, scores were generally higher (median 2, range 1–2), particularly for outcome assessment, although all studies 28 (100%) relied exclusively on self-reported measures; reporting of statistical tests was clearly described and appropriate in most studies 22 (79%).^32^, ^33^, ^34^, ^35^^,^^37^, ^38^, ^39^, ^40^, ^41^, ^42^, ^43^, ^44^, ^45^, ^46^, ^47^, ^48^^,^^53^, ^54^, ^55^, ^56^, ^57^, ^58^ Study quality assessments are summarized in eTable 3.

### Prevalence of PrEP use among adult cisgender GBMSM engaging in chemsex

**Fig. 2:**
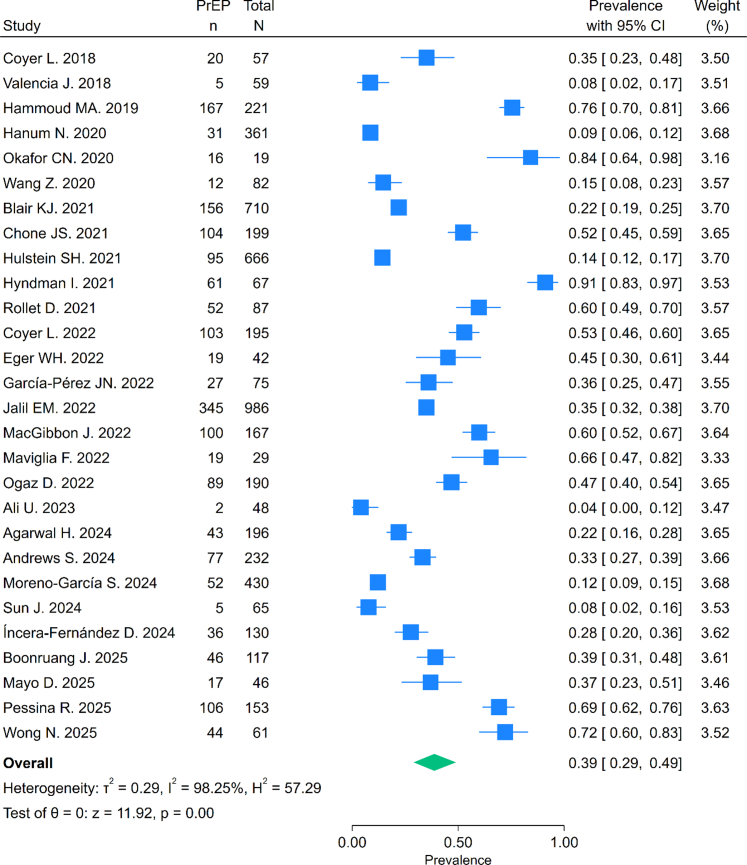
**Forest plot of prevalence of PrEP use in the overall analysis. CI: Confidence Intervals. PrEP: Pre-Exposure Prophylaxis**.

**Fig. 3:**
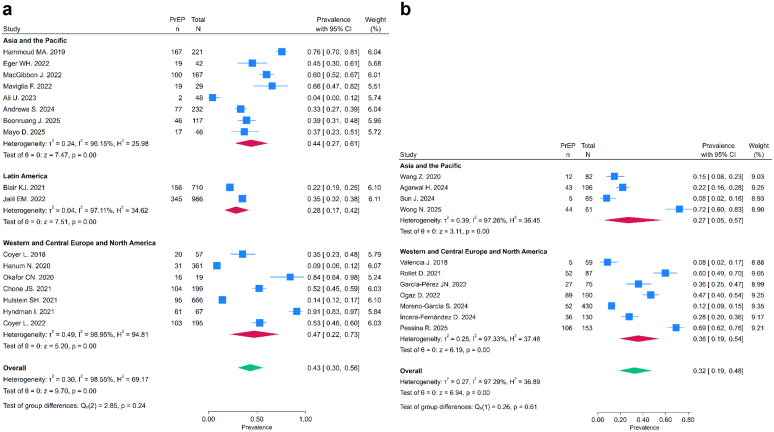
**Forest plot of prevalence of PrEP use by subgroups**. a: Forest plot of PrEP use prevalence among adult cisgender GBMSM with *recent* chemsex engagement (less than 6 months). b: Forest plot of PrEP use prevalence among adult cisgender GBMSM with *ever* chemsex engagement. CI: Confidence Intervals. PrEP: Pre-Exposure Prophylaxis. GBMSM: Gay, bisexual and other men who have sex with men.

The pooled prevalence of PrEP use was 39% (95% CI: 29–49%) (Fig. 2). We found no statistically significant difference between *recent* chemsex engagement with *ever* timeframes of engagement (Fig. 3). The leave-one-out sensitivity analysis indicated that no single study substantially influenced the pooled prevalence estimates (eFig. 1). Meta-regression analyses for *recent* and *ever* chemsex exposure timeframes and study year, country income group, or world region found no statistical difference in the prevalence of PrEP use (p > 0.05), and these covariates explained very little of the between-study variance. Heterogeneity remained high (I^2^ > 97%) across all analyses (eTable 5 and eTable 6).

### Chemsex and odds of PrEP use among adult cisgender GBMSM

**Fig. 4:**
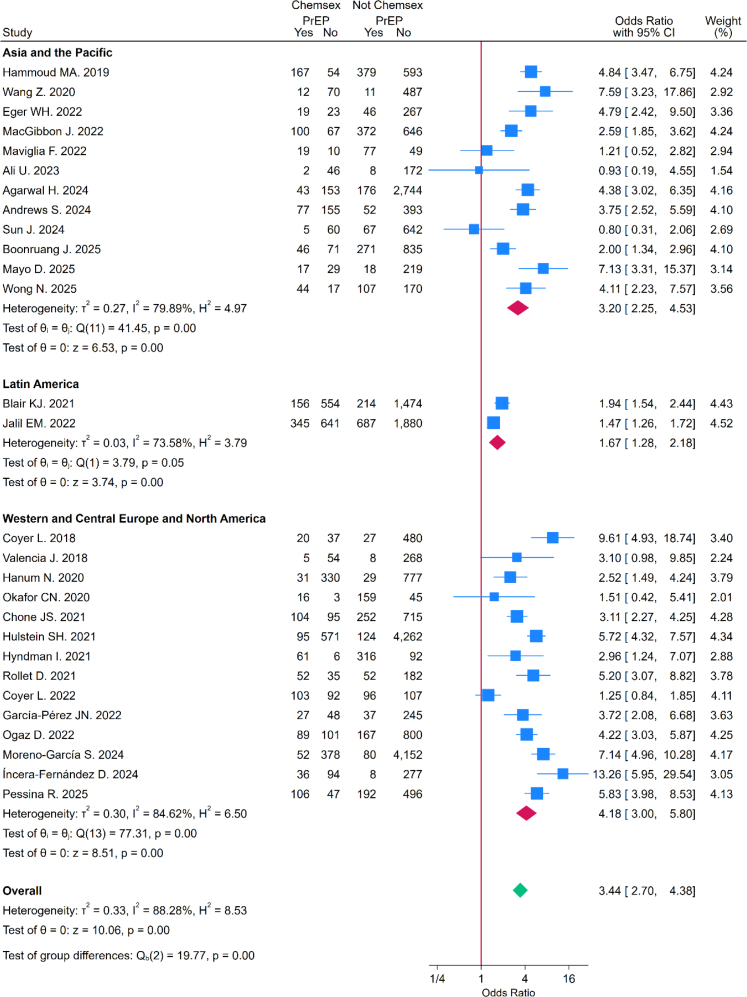
**Forest Plot for overall association between chemsex and PrEP use stratified by world regions**. This forest Plot shows the overall association between chemsex practice (*recent* and *ever*) and PrEP use stratified by UNAIDS world regions. CI: Confidence Intervals. PrEP: Pre-Exposure Prophylaxis. UNAIDS: The Joint United Nations Program on HIV/AIDS.

**Fig. 5:**
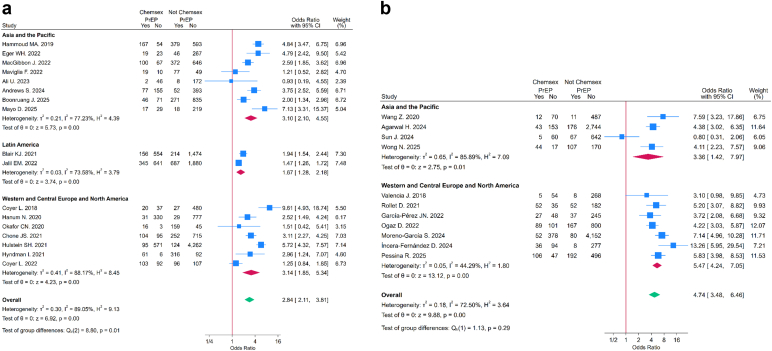
**Forest plot for the association between chemsex and PrEP use stratified by chemsex practice timeframe (*recent*/*ever*) and world regions**. a: Forest plot showing the association between *recent* chemsex practice and PrEP use stratified by UNAIDS world regions. b: Forest plot showing the association between *ever* chemsex practice and PrEP use stratified by UNAIDS world regions. CI: Confidence Intervals. PrEP: Pre-Exposure Prophylaxis. UNAIDS: The Joint United Nations Program on HIV/AIDS.

**Fig. 6:**
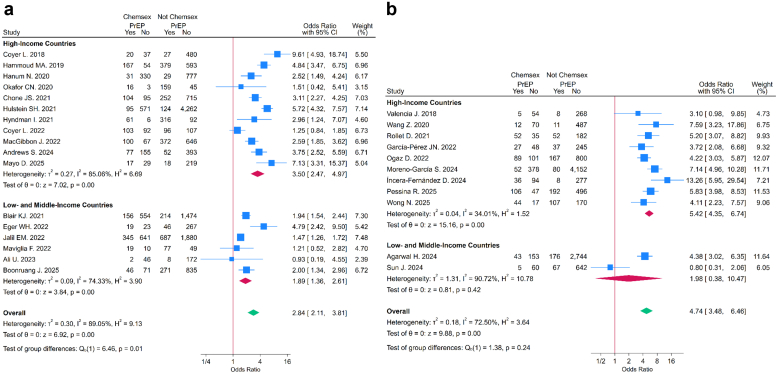
**Forest plot for the association between chemsex use and PrEP use stratified by chemsex use timeframe (recent/ever) and country income classification**. a: Forest plot showing the association between recent chemsex exposure and PrEP use stratified by country income classification. b: Forest plot showing the association between ever chemsex exposure and PrEP use stratified country income classification. CI: Confidence Intervals. PrEP: Pre-Exposure Prophylaxis.

The pooled OR indicated that chemsex exposure was associated with higher odds of PrEP use (OR = 3.44, 95% CI: 2.70–4.38) (Fig. 4). In subgroup analyses by timing of chemsex engagement, the association with PrEP use was greater among those reporting *ever* chemsex (OR = 4.74, 95% CI: 3.48–6.46) compared with those reporting *recent* chemsex (OR = 2.84, 95% CI: 2.11–3.81). This difference was statistically significant (test for subgroup differences: p = 0.019) (Fig. 5), (eTable 7). The leave-one-out analysis indicated that no single study substantially influenced the pooled ORs (eFig. 2). The meta-regressions with country income group as moderator were statistically significant for both *recent* (OR = 0.54, 95% CI: 0.31–0.94; p = 0.031) and *ever* chemsex timeframes (OR = 0.46, 95% CI: 0.21–0.99; p = 0.046), indicating that in LMICs, the association between chemsex and PrEP uptake was weaker compared to HICs (Fig. 6) (eTable 8 and eTable 9). None of the other moderators were statistically significant in either group (p > 0.05), and they explained little of the between-study variance. Heterogeneity remained moderate to high (I^2^ > 70%) across all analyses.

### Small-study effects and publication bias

The PrEP use prevalence meta-analyses showed minor asymmetry for the overall estimate (eFig. 3) and major asymmetry for both *recent* and *ever* chemsex engagement (eFig. 4), suggesting possible small-study effects or selective publication bias, particularly in the time-specific subgroups. In contrast, the chemsex–PrEP association meta-analyses showed no evidence of small-study effects or publication bias, either in the overall analysis (eFig. 5) or in the time-specific subgroups (eFig. 6).

## Discussion

This is the first systematic review and meta-analysis to provide a global summary of the interplay between chemsex practice and PrEP use in adult cisgender GBMSM, addressing a critical gap in the literature to optimize HIV prevention in this population. A total of 28 studies, including 36,339 participants, from three world regions were collated. Across all studies, 39% of GBMSM who engaged in chemsex reported the use of PrEP. This estimate is higher than the 11–16% PrEP use reported in a previous global meta-analysis^60^ of MSM, although differences in study populations, definitions, and lack of disaggregated data in earlier studies limit direct comparison, as that review did not specifically evaluate adult cisgender GBMSM who engage in chemsex. GBMSM who reported engagement in chemsex were over three times more likely to report the use of PrEP compared to those who did not engage in chemsex. Additionally, studies conducted in LMICs reported significantly lower odds of PrEP use in this group compared with those in HICs.

With respect to chemsex timeframes, the association with PrEP use was stronger among individuals reporting *ever* chemsex than *recent* engagement, which may partly reflect the longer exposure window captured by *ever* chemsex. A number of factors may play a role in this result, determining a shift in the risk perception or access to PrEP services. Individuals with past or unspecified chemsex engagement may have had more opportunities to initiate PrEP or may represent a subgroup with more sustained engagement in healthcare. This association may also reflect contextual factors, including greater health-seeking behavior among some individuals who engage in chemsex and the wider availability of PrEP in urban sexual health services where chemsex is more frequently reported, although the cross-sectional evidence does not allow these influences to be disentangled. Conversely, those reporting *recent* chemsex may face more acute barriers to PrEP access, or less perception of risk. Despite the robust association observed, the published studies are limited to local surveys with high heterogeneity across them, such as differences in study design, population characteristics, definitions of chemsex, and healthcare systems. These results therefore need to be interpreted with caution, as they reflect studies published between 2018 and July 2025, a period during which PrEP availability and access varied substantially across countries and regions and remained below global targets.^19^ Similarly, the lower odds of PrEP use in GBMSM observed in studies from LMICs may be a result by legal restrictions, health system challenges, and social barriers.^61^ In settings where laws criminalize same–sex relations or drug use can discourage individuals from seeking care, limiting access to PrEP, and obscuring the reach of these practices. Chemsex is associated with high-risk sexual behaviors and drug use patterns, that can lead to severe sexual and mental health and social consequences. Studies among GBMSM living with HIV^62^^,^^63^ report poorer treatment adherence among individuals engaging in chemsex or SDU. Although these findings concern HIV treatment rather than prevention, they highlight behavioral and psychosocial factors that may similarly impede consistent PrEP use. Motivations described for the practice range from pleasure-seeking to coping with stigma and emotional distress, with psychosocial factors strongly linked to problematic patterns and adverse outcomes.^12^, ^13^, ^14^ In this regard, the conceptual framework proposed in *The problematic chemsex journey (2019)*^64^ describes a six-stage progression from feelings of loneliness and emptiness to problematic chemsex. This model emphasizes the importance of addressing both behavioral and psychosocial dimensions to prevent escalation and guide effective interventions highlighting the importance of interventions addressing both behavioral and psychosocial dimensions. Providing services to GBMSM who engage in chemsex practices remains a global challenge that needs addressing stigma, cultural sensitivities, financial and structural barriers, strengthening health workers’ capacity, infectious disease prevention (including hepatitis C), harm reduction, and mental health care,^65^ especially in LMICs. Conventional drug services, largely designed for opioid or alcohol dependence, often lack cultural competence and relevance for GBMSM, underscoring the importance of multidisciplinary approaches in partnership with community organizations and peers. A recent systematic review of harm reduction strategies^66^ revealed that interventions for chemsex among MSM include a web-based programs, peer-led support, and mobile health services. Digital interventions, such as mindfulness-based cognitive approaches and harm reduction platforms, improve self-efficacy and promote safer practices, while peer-led programs, particularly those facilitated by individuals with lived experience, demonstrate greater engagement and abstinence rates. Mobile health initiatives, such as distributing safer sex and drug use kits, have also proven beneficial. While some GBMSM engaging in chemsex may use PrEP as part of proactive health-seeking behavior, PrEP alone does not address the broader physical, psychological, and social harms associated with chemsex. In addition, a recent study^67^ describes the implementation of a multidisciplinary approach for people living with HIV who engage in chemsex, showing a positive impact on substance use and enabling the identification of unmet needs and vulnerable subgroups, such as migrants, individuals who practice slam, and sex workers, who still require targeted interventions. HIV, sexual health, and harm reduction services should routinely incorporate PrEP and chemsex care. In this context, the 2025 WHO guidelines expanding the HIV prevention toolkit with long-acting injectable lenacapavir, administered only twice yearly, represent a transformative step for GBMSM at risk of HIV, particularly those facing challenges with daily adherence, stigma, or limited healthcare access.^68^ Despite the overall variability, our findings were consistent across settings and world regions, suggesting that the association between chemsex and PrEP uptake among adult cisgender GBMSM is not context-specific but a global phenomenon.

Studies lacked detailed descriptions of chemsex use and behavioral patterns, such as frequency, dose, or mode of administration (e.g., intravenous use or *slamsex*).^58^ The timeframe of the use of chemsex was often loosely categorized and was reported by the study participants. Similarly, the definitions of PrEP use across included studies were also heterogeneous, ranging from *current* reported use to *ever* PrEP use, and, in some cases, informal^40^ or off-prescription use.^53^ All studies reported oral PrEP use, and only one study reported injectable PrEP with cabotegravir.^46^ This reflects the evolution of clinical guidelines and the historical availability of PrEP formulations for this group.^16^ The lack of standardized definitions and variability across studies limit the ability to fully understand the interplay between chemsex and PrEP use in GBMSM,^69^ considering that different chemsex patterns may shape prevention needs and affect PrEP uptake and continuation in this group.^70^

This review has several strengths, including a comprehensive search across major databases without language or time restrictions, systematic literature quality assessment, and the use of subgroup, sensitivity, and meta-regression analyses. However, important limitations should be considered. Although the search was comprehensive, relevant studies may have been missing if not indexed or unpublished. All included studies were observational, cross-sectional reports, which preclude establishing causality or temporality, and their overall methodological quality was low to moderate. Reliance on self-reported information regarding use of chemsex or PrEP in participants introduces risks of recall error and social desirability bias. Variability in measurement of chemsex and PrEP definitions reduced comparability across studies and likely contributed to the heterogeneity observed. In addition, evidence of possible small-study effects or publication bias was detected in the prevalence analyses, which may have led to overestimation of pooled prevalence. In many reports, the odds of PrEP use among chemsex-engaged GBMSM were presented as secondary findings, increasing the risk of selective reporting. Because the included studies span multiple years and a limited number of countries, our pooled prevalence should not be interpreted as reflecting current global PrEP uptake, and temporal and regional variability further limits generalizability. Geographic representation was uneven, with greater weight from high-income settings and no studies from regions with a high HIV burden, such as sub-Saharan Africa, limiting the generalizability, and highlighting the need to generate global evidence in the field. In particular, estimates for LA and for AP were derived from a small number of studies, and should therefore be interpreted with caution. More fundamentally, the number of studies that report both chemsex and PrEP uptake remains very limited globally, which reflects a structural evidence gap rather than a search limitation, as relevant information may exist only in gray literature, which was excluded. Moreover, our review focused on estimating prevalence and associations rather than characterizing individual profiles of GBMSM engaging in chemsex, as most included studies provided limited disaggregated data beyond exposure and outcome measures. While these limitations warrant caution, in a context where randomized trials are not feasible, this synthesis of standardized observational evidence becomes essential to inform practice and global policy recommendations.

Important evidence gaps remain.^71^ Future studies should adopt standardized definitions of chemsex and PrEP to improve comparability, with consensus on a core set of indicators including standard terminology, substance descriptions, frequency, patterns, and timeframe of chemsex exposure, as well as standardized reporting of PrEP options, including initiation and continuation of PrEP use.^72^ Additionally, future research should prioritize prospective longitudinal studies to clarify the temporal relationship between chemsex and PrEP initiation and continuation. Qualitative and mixed-methods research is needed to capture lived experiences, motivations, and barriers that may explain heterogeneity across settings and will be critical to strengthen the evidence base in the field. Research should also move beyond prevalence estimates to better characterize GBMSM engaging in chemsex, considering age, migration status, and socioeconomic position. Age-stratified analyses, in particular, would be valuable to capture differences in patterns of chemsex practice and prevention needs across the course of life. Other populations with distinct vulnerabilities, patterns of chemsex practice, and different barriers or facilitators to prevention, such as transgender people and minor GBMSM, also warrant dedicated investigation.^73^ Finally, geographic coverage for evidence generation must be extended to underrepresented regions, as current evidence comes predominantly from high-income settings, with significant areas missing, such as sub-Saharan Africa and the Middle East and North Africa.^74^ Scaling up integrated PrEP provision with comprehensive harm reduction and broader health promotion efforts for GBMSM across settings and cultures, with particular attention to LMICs, can significantly improve health outcomes for this population and contribute to the global HIV elimination goals.

## Contributors

JGM and LGF conceived the idea for the review, designed and coordinated the study. JGM, SAG, and LGF acquired data, screened records, extracted data, and assessed the quality of the evidence. EDL conducted data analysis. JGM, SAG, and LGF wrote the first draft of the manuscript. EDL, FJMC, LM, ML, JM, MMR, and AC gave crucial intellectual input and provided critical revisions in the manuscript. JGM, SAG and LGF have verified the underlying data and had the final responsibility for the decision to submit for publication. All authors had full access to all the data in the study and approved the final version of the manuscript. JGM and LGF managed the project timelines and coordinated tasks among the research team.

## Data sharing statement

The data that support the findings of this study are available from the corresponding author upon reasonable request.

## Declaration of interests

ML has received institutional research funding and speaker honoraria from ViiV Healthcare, Gilead Sciences, and Janssen, and has participated in advisory boards for Janssen and Gilead Sciences.

JM has received payments for expert testimony from MSD, ViiV Healthcare, Gilead Sciences, and Janssen, and the HIV Unit in which he works has received institutional research funding from these companies.

AC works in an HIV Unit that receives institutional funding for industry-sponsored trials from ViiV Healthcare, MSD, and Gilead Sciences.

All other authors declare no competing interests.
