# Supplementary material for: Chemsex and uptake of HIV pre-exposure prophylaxis among adult cisgender gay, bisexual and other men who have sex with men worldwide: a systematic review with meta-analysis
Source: eClinicalMedicine. 2026 Mar 5;93:103804. doi: 10.1016/j.eclinm.2026.103804 (PMC13043317; doi:10.1016/j.eclinm.2026.103804)
Supplement: Supplementary Figures and Tables [file mmc1.docx]

**Supplementary materials**

**Title:** Chemsex and uptake of HIV pre-exposure prophylaxis among adult cisgender gay, bisexual and other men who have sex with men worldwide: a systematic review with meta-analysis

**Authors:** Joan Gil Miñana, Sonia Arias García, Elisa De Lazzari, Francisco José Montoya Conesa, Lorena De La Mora, Montserrat Laguno, Josep Mallolas, Maria Martínez Rebollar, Alexandra Calmy, Lucía González Fernández.

**Table of Contents**

[eTable 1. PICO concepts 2](#_Toc215913533)

[eTable 2: Literature search Strategy 3](#_Toc215913534)

[eTable 3. Quality assessment of included studies 6](#_Toc215913535)

[eTable 4: Reason for studies exclusion at full text review 7](#_Toc215913536)

[eFigure 1. Leave-one-out analysis of PrEP use prevalence by subgroups 10](#_Toc215913537)

[eTable 5. Meta-regression of PrEP Use Prevalence (recent chemsex engagement) 11](#_Toc215913538)

[eTable 6. Meta-regression of PrEP Use Prevalence (ever chemsex engagement) 12](#_Toc215913539)

[eTable 7. Meta-regression comparing recent vs. ever chemsex engagement: odds ratios 13](#_Toc215913540)

[eFigure 2. Leave-one-out analysis of chemsex use by subgroups 14](#_Toc215913541)

[eTable 8. Meta-regression of the association between chemsex and PrEP use (recent chemsex engagement): odds ratios 15](#_Toc215913542)

[eTable 9. Meta-regression of the association between chemsex and PrEP use (ever chemsex engagement): odds ratios 16](#_Toc215913543)

[eFigure 3. DOI plot assessing publication bias in the overall analysis 17](#_Toc215913544)

[eFigure 4. DOI plot assessing publication bias by subgroups 18](#_Toc215913545)

[eFigure 5. Funnel plot assessing publication bias in the overall analysis 19](#_Toc215913546)

[eFigure 6. Funnel plot assessing publication bias by subgroups 20](#_Toc215913547)

| **eTable 1. PICO concepts** | |
| --- | --- |
| **Population** | HIV-negative cis-gender men aged 18 years or older who identify as GBMSM worldwide |
| **Intervention/ Exposure** | Chemsex practice (“recent” or” ever”, as reported by the authors). Substances include those that can induce psychotropic, pain-relieving, Psychedelic, or hallucinogenic effects, such as crystal methamphetamine, mephedrone, GHB, gamma-butyrolactone, ecstasy, cocaine, amyl nitrites (poppers), ketamine, heroin, alcohol, marijuana, sildenafil nitrate, and other erectile dysfunction drugs. Or others, as described by the authors, taken with the intention to influence sexual experiences |
| **Comparators** | Absence of chemsex practice |
| **Outcomes** | Uptake of HIV any PrEP, either daily, on demand oral or injectable options |

# **eTable 2: Literature search Strategy**

| **Database:** | **Medline (via Ovid)** |
| --- | --- |
| **Date:** | **4 December 2024** |
|  | **1 July 2025** |
| **No.** | **Query** |
| 1 | homosexuality, male/ or bisexuality/ or homosexuality/ or "Sexual and Gender Minorities"/ or ((men or man or male) adj2 ("sex with men" or "same sex" or gay or queer or homosexual or bisexual or "sexual minority")).ti,ab,kw. or (MSM or GBM or SMM or SGM).ti,ab,kw. |
| 2 | (exp Sexual Behavior/ and (exp Substance-Related Disorders/ or exp illicit drugs/ or "recreational drug use"/)) or ((sex or sexualized or sexual or intercourse) adj3 (chemical or substance or drug* or slam or stimulant* or PnP)).ti,ab,kw. or (chemsex or "party and play").ti,ab,kw. |
| 3 | Pre-Exposure Prophylaxis/ or (exp *HIV Infections/pc and exp Anti-HIV Agents/) or ("pre-exposure prophylaxis" or prep).ti,ab,kw. or ((HIV or "human immunodeficiency virus" or ARV or antiretroviral) adj6 (prophyla* or prevent*)).ti,ab,kw. |
| 4 | 1 and 2 and 3 |
| 5 | 4 not (animals not humans).sh. |
| 6 | 5 not ((exp child/ or exp infant/ or exp adolescent/) not exp adult/) |
| 7 | limit 6 to (congress or preprint) |
| 8 | 6 not 7 |

| **Database:** | **Embase (via Elsevier)** |
| --- | --- |
| **Date:** | **4 December 2024** |
|  | **1 July 2025** |
| **No.** | **Query** |
| #1 | 'men who have sex with men'/exp OR 'men who have sex with men and women'/de OR 'homosexual male'/de OR 'male homosexuality'/exp OR 'sexual and gender minority'/de OR 'lgbtqia+ people'/de OR 'lgbt people'/de OR 'bisexual male'/de OR (((men OR man OR male) NEAR/2 ('sex with men' OR 'same sex' OR gay OR queer OR homosexual OR bisexual OR 'sexual minority')):ti,ab,kw) OR msm:ti,ab,kw OR gbm:ti,ab,kw OR smm:ti,ab,kw OR sgm:ti,ab,kw |
| #2 | 'sexualized drug use'/exp OR 'chemsex'/exp OR ('sexual behavior'/exp AND ('drug use'/de OR 'recreational drug use'/exp OR 'substance use'/exp)) OR (((sex OR sexualized OR sexual OR intercourse) NEAR/3 (chemical OR substance OR drug* OR slam OR stimulant* OR pnp)):ti,ab,kw) OR chemsex:ti,ab,kw OR 'party and play':ti,ab,kw |
| #3 | 'pre-exposure prophylaxis'/exp OR ('anti human immunodeficiency virus agent'/exp AND 'prophylaxis'/exp) OR 'pre-exposure prophylaxis':ti,ab,kw OR prep:ti,ab,kw OR (((hiv OR 'human immunodeficiency virus' OR arv OR antiretroviral) NEAR/6 (prophyla* OR prevent*)):ti,ab,kw) |
| #4 | #1 AND #2 AND #3 |
| #5 | #4 NOT (('animal'/de OR 'animal experiment'/exp OR 'nonhuman'/de) NOT ('human'/exp OR 'human experiment'/de)) |
| #6 | #5 NOT (([infant]/lim OR [child]/lim OR [adolescent]/lim) NOT ([adult]/lim OR [aged]/lim)) |
| #7 | #6 NOT ([conference abstract]/lim OR [preprint]/lim) |

| **Database:** | **Cochrane Reviews and CENTRAL (via Cochrane Library / Wiley)** |
| --- | --- |
| **Date:** | **4 December 2024** |
|  | **1 July 2025** |
| **No.** | **Query** |
| 1 | ((men or man or male) NEAR/2 ("sex with men" OR "same sex" OR gay or queer or homosexual or bisexual OR "sexual minority")):ti,ab,kw OR (MSM OR GBM OR SMM OR SGM):ti,ab,kw |
| 2 | ((sex OR sexualized OR sexual OR intercourse) NEAR/3 (chemical OR substance OR drug* OR slam OR stimulant* OR PnP)):ti,ab,kw OR (chemsex OR "party and play"):ti,ab,kw |
| 3 | ("pre-exposure prophylaxis" OR prep):ti,ab,kw OR ((HIV OR "human immunodeficiency virus" OR ARV OR antiretroviral) NEAR/6 (prophyla* OR prevent*)):ti,ab,kw |
| 4 | #1 AND #2 AND #3 |

| **Database:** | **APA PsycInfo (via EBSCOhost)** |
| --- | --- |
| **Date:** | **4 December 2024** |
|  | **1 July 2025** |
| **No.** | **Query** |
| S1 | DE "LGBTQ" OR DE "Bisexuality" OR DE "Male Homosexuality" OR DE "Sexual Minority Groups" OR DE "Homosexuality" OR TI((men or man or male) N2 ("sex with men" OR "same sex" OR gay or queer or homosexual or bisexual OR "sexual minority")) OR AB((men or man or male) N2 ("sex with men" OR "same sex" OR gay or queer or homosexual or bisexual OR "sexual minority")) OR KW((men or man or male) N2 ("sex with men" OR "same sex" OR gay or queer or homosexual or bisexual OR "sexual minority")) OR TI(MSM OR GBM OR SMM OR SGM) OR AB(MSM OR GBM OR SMM OR SGM) OR KW(MSM OR GBM OR SMM OR SGM) |
| S2 | ((DE "Psychosexual Behavior" OR DE "Sexual Risk Taking" OR DE "Same Sex Intercourse" OR DE "Sexual Intercourse (Human)" OR DE "Sexual Aids" OR DE "Sexual Arousal") AND (DE "Drugs" OR DE "Drug Usage" OR DE "Drug Abuse" OR DE "Polydrug Abuse" OR DE "Substance Use Disorder" OR DE "Drug Addiction" OR DE "Self-Medication")) OR TI((sex OR sexualized OR sexual OR intercourse) N3 (chemical OR substance OR drug* OR slam OR stimulant* OR PnP)) OR AB((sex OR sexualized OR sexual OR intercourse) N3 (chemical OR substance OR drug* OR slam OR stimulant* OR PnP)) OR KW((sex OR sexualized OR sexual OR intercourse) N3 (chemical OR substance OR drug* OR slam OR stimulant* OR PnP)) OR TI(chemsex OR "party and play") OR AB(chemsex OR "party and play") OR KW(chemsex OR "party and play") |
| S3 | DE "Pre-Exposure Prophylaxis" OR DE "AIDS Prevention" OR TI("pre-exposure prophylaxis" OR prep) OR AB("pre-exposure prophylaxis" OR prep) OR KW("pre-exposure prophylaxis" OR prep) OR TI((HIV OR "human immunodeficiency virus" OR ARV OR antiretroviral) N6 (prophyla* OR prevent*)) OR AB((HIV OR "human immunodeficiency virus" OR ARV OR antiretroviral) N6 (prophyla* OR prevent*)) OR KW((HIV OR "human immunodeficiency virus" OR ARV OR antiretroviral) N6 (prophyla* OR prevent*)) |
| S4 | S1 AND S2 AND S3 |
| S5 | S4 NOT ((DE "Animal Models" OR DE "Animals" OR DE "Animal Research" OR TI (animal model*)) NOT (DE "Human Males" OR DE "Human Females")) |
| S6 | S5 NOT ((DE "Childhood Development" OR DE "Adolescent Development" OR DE "Early Adolescence" OR DE "Late Adolescence" OR DE "Puberty") NOT (DE "Adult Development" OR DE "Emerging Adulthood" OR DE "Middle Adulthood" OR DE "Older Adulthood")) |
| S7 | S6 Limiters - Exclude Dissertations |

| **Database:** | **Scopus (via Elsevier)** |
| --- | --- |
| **Date:** | **4 December 2024** |
|  | **1 July 2025** |
| **No.** | **Query** |
| 1 | TITLE-ABS-KEY((men or man or male) W/2 ("sex with men" OR "same sex" OR gay or queer or homosexual or bisexual OR "sexual minority")) OR TITLE-ABS-KEY(MSM OR GBM OR SMM OR SGM) |
| 2 | TITLE-ABS-KEY((sex OR sexualized OR sexual OR intercourse) W/3 (chemical OR substance OR drug* OR slam OR stimulant* OR PnP)) OR TITLE-ABS-KEY(chemsex OR "party and play") |
| 3 | TITLE-ABS-KEY("pre-exposure prophylaxis" OR prep) OR TITLE-ABS-KEY((HIV OR "human immunodeficiency virus" OR ARV OR antiretroviral) W/6 (prophyla* OR prevent*)) |
| 4 | #1 AND #2 AND #3 |

| **Database:** | **LILACS (via VHL Regional Portal)** |
| --- | --- |
| **Date:** | **4 December 2024** |
|  |  |
| **No.** | **Query** |
| 1 | (((men OR man OR male) AND ("sex with men" OR "same sex" OR gay OR queer OR homosexual OR bisexual OR "sexual minority")) OR (msm OR gbm OR smm OR sgm)) AND (((sex OR sexualized OR sexual OR intercourse) AND (chemical OR substance OR drug* OR slam OR stimulant* OR pnp)) OR (chemsex OR "party and play")) AND (("pre-exposure prophylaxis" OR prep) OR ((hiv OR "human immunodeficiency virus" OR arv OR antiretroviral) AND (prophyla* OR prevent*))) AND instance:"regional" |
| 2 | #1 AND ( db:("LILACS")) |

# **eTable 3. Quality assessment of included studies**

|  | | | | | | | | | | | |
| --- | --- | --- | --- | --- | --- | --- | --- | --- | --- | --- | --- |
| Study  Author and publication year | Selection | | | | Comparability | Outcome | | | Score | | Quality |
|  | 1 | 2 | 3 | 4 |  | 1 | 2 |  | |  | |
| Coyer et al. 2018 | **✵** | **✵** | **-** | **-** | **-** | **✵** | **✵** | 4 | | Medium | |
| Valencia et al. 2018 | **✵** | **✵** | **✵** | **-** | **-** | **✵** | **✵** | 5 | | Medium | |
| Hammoud et al. 2019 | **-** | **✵** | **-** | **✵** | **-** | **✵** | **✵** | 4 | | Medium | |
| Hanum et al. 2020 | **✵** | **✵** | **-** | **✵** | **-** | **✵** | **✵** | 5 | | Medium | |
| Okafor et al. 2020 | **✵** | **-** | **-** | **✵** | **-** | **✵** | **-** | 3 | | Low | |
| Wang et al. 2020 | **✵** | **✵** | - | **✵** | **✵** | **✵** | **✵** | 6 | | Medium | |
| Blair et al. 2021 (Brazil) | - | **✵** | - | - | - | **✵** | **✵** | 3 | | Low | |
| Chone et al. 2021 | **-** | **✵** | **-** | - | **✵** | **✵** | **✵** | 4 | | Medium | |
| Hulstein et al. 2021 | **✵** | **✵** | - | **✵** | - | **✵** | **✵** | 5 | | Medium | |
| Hyndman et al. 2021 | - | **✵** | - | - | - | **✵** | **✵** | 3 | | Low | |
| Rollet et al. 2021 | **✵** | **-** | **-** | **✵** | **✵** | **✵** | **✵** | 5 | | Medium | |
| Coyer et al. 2022 | **✵** | **✵** | **-** | **✵** | **-** | **✵** | **✵** | 5 | | Medium | |
| Eger et al. 2022 | **-** | **-** | **-** | **-** | **-** | **✵** | **✵** | 2 | | Low | |
| García-Pérez et al. 2022 | **✵** | **✵** | **-** | **-** | **✵** | **✵** | **✵** | 5 | | Medium | |
| Jalil et al. 2022 | **✵** | **✵** | **-** | **✵** | **✵** | **✵** | **✵** | 6 | | Medium | |
| MacGibbon et al. 2022 | **-** | **✵** | **-** | **✵** | **-** | **✵** | **✵** | 4 | | Medium | |
| Maviglia et al. 2022 | **-** | **✵** | **-** | **✵** | **✵** | **✵** | **✵** | 5 | | Medium | |
| Ogaz et al. 2022 | **✵** | **✵** | **-** | **✵** | **-** | **✵** | **-** | 4 | | Medium | |
| Ali et al. 2023 | **-** | **-** | **-** | **-** | **-** | **✵** | **-** | 1 | | Low | |
| Agarwal et al. 2024 | **-** | **✵** | **-** | **✵** | **-** | **✵** | **-** | 3 | | Low | |
| Andrews et al. 2024 | **-** | **✵** | **-** | **-** | **✵** | **✵** | - | 3 | | Low | |
| Íncera-Fernández et al. 2024 | **-** | **✵** | **-** | **✵** | **✵** | **✵** | **✵** | 5 | | Medium | |
| Moreno-García et al. 2024 | **-** | **✵** | **-** | **✵** | **-** | **✵** | **✵** | 4 | | Medium | |
| Sun et al. 2024 | **✵** | **✵** | **-** | **✵** | **✵** | **✵** | **✵** | 6 | | Medium | |
| Boonruang et al. 2025 | **✵** | **✵** | **✵** | **✵** | **-** | **✵** | **✵** | 6 | | Medium | |
| Mayo et al. 2025 | **-** | **-** | **-** | **✵** | **-** | **✵** | **✵** | 3 | | Low | |
| Pessina et al. 2025 | **-** | **✵** | **-** | **✵** | **-** | **✵** | **✵** | 4 | | Medium | |
| Wong et al. 2025 | **-** | **-** | **-** | **-** | **✵** | **✵** | **-** | 2 | | Low | |

#

The assessment was conducted using the Adapted Newcastle-Ottawa Scale for Cross-Sectional Studies (<https://cdn-links.lww.com/permalink/ejgh/a/ejgh_31_9_2019_07_18_nguyen_15743_sdc1.pdf> ). The items marked with a star (*) for each category are counted as one (“1”) and factored into the final scoring which ranges from zero to nine (lowest to highest). To simplify interpretation those studies that scored 7 or above were categorized as high quality, those scoring between 4-6 were of moderate quality, and those scoring below 4 were considered low quality.

# **eTable 4: Reason for studies exclusion at full text review**

| **Study** | **Reasons for exclusion** |
| --- | --- |
| Adamson 2025 | Relationship between exposure and outcome not directly addressed |
| Ahaus 2022 | Do not describe the desire exposure and outcome |
| Ambrozic 2024 | Do not describe the desire exposure and outcome |
| Bavinton 2020 | Relationship between exposure and outcome not directly addressed |
| Beyrer 2017 | Inappropriate study design |
| Blair 2021 | Population other than described in PICO question |
| Blair 2022 | Incomplete data |
| Brogan 2019 | Relationship between exposure and outcome not directly addressed |
| Callander 2019 | Relationship between exposure and outcome not directly addressed |
| Chai 2022 | Drug use other than chemsex |
| Chan 2022 | Relationship between exposure and outcome not directly addressed |
| Chow 2021 | Do not describe the desire exposure and outcome |
| Chow 2021 | Do not describe the desire exposure and outcome |
| Closson 2018 | Do not describe the desire exposure and outcome |
| Costa 2025 | Incomplete data |
| Coyer 2020 | Do not describe the desire exposure and outcome |
| Dai 2019 | Do not describe the desire exposure and outcome |
| Dai 2024 | Incomplete data |
| Dai 2025 | Do not describe the desire exposure and outcome |
| Dangerfield 2021 | Relationship between exposure and outcome not directly addressed |
| De la Court 2023 | Do not describe the desire exposure and outcome |
| DeLaMora 2022 | Do not describe the desire exposure and outcome |
| DelPozo-Herce 2024 | Incomplete data |
| Du 2025 | Incomplete data |
| Dubov 2018 | Do not describe the desire exposure and outcome |
| Eaton 2018 | Do not describe the desire exposure and outcome |
| Ertl 2025 | Do not describe the desire exposure and outcome |
| Feinstein 2018 | Do not describe the desire exposure and outcome |
| Feinstein 2019 | Relationship between exposure and outcome not directly addressed |
| Feldman 2020 | Drug use other than chemsex |
| Flores Anato 2021 | Do not describe the desire exposure and outcome |
| Flores Anato 2022 | Do not describe the desire exposure and outcome |
| Foucha 2022 | Do not describe the desire exposure and outcome |
| Fusetti 2025 | Population other than described in PICO question |
| Gafos 2019 | Do not describe the desire exposure and outcome |
| Gerke 2022 | Relationship between exposure and outcome not directly addressed |
| Gibson 2022 | Relationship between exposure and outcome not directly addressed |
| Goodman-Meza 2019 | Inappropriate study design |
| Gras 2021 | Inappropriate study design |
| Gras 2022 | Relationship between exposure and outcome not directly addressed |
| Gras 2023 | Do not describe the desire exposure and outcome |
| Grov 2019 | Drug use other than chemsex |
| Hage 2024 | Do not describe the desire exposure and outcome |
| Hammond 2023 | Incomplete data |
| Hammoud 2018 | Do not describe the desire exposure and outcome |
| Hammoud 2020 | Relationship between exposure and outcome not directly addressed |
| Hanum 2021 | Relationship between exposure and outcome not directly addressed |
| Hanum 2025 | Incomplete data |
| Hardy 2022 | Incomplete data |
| Hibbert 2019 | Incomplete data |
| Hibbert 2020 | Population other than described in PICO question |
| Holtz 2016 | Inappropriate study design |
| Hoornenborg 2018 | Do not describe the desire exposure and outcome |
| Horton 2024 | Do not describe the desire exposure and outcome |
| Hovaguimian 2022 | Do not describe the desire exposure and outcome |
| Jain 2021 | Relationship between exposure and outcome not directly addressed |
| Javanbakht 2023 | Relationship between exposure and outcome not directly addressed |
| Kamadjou 2024 | Do not describe the desire exposure and outcome |
| Khaw 2021 | Population other than described in PICO question |
| Knox 2020 | Relationship between exposure and outcome not directly addressed |
| Kwan 2019 | Do not describe the desire exposure and outcome |
| Laguno 2023 | Do not describe the desire exposure and outcome |
| Latini 2019 | Relationship between exposure and outcome not directly addressed |
| Liu 2013 | Do not describe the desire exposure and outcome |
| Logan 2019 | Incomplete data |
| L'Yavanc 2023 | Incomplete data |
| MacGregor 2021 | Relationship between exposure and outcome not directly addressed |
| Mansergh 2020 | Incomplete data |
| Marcus 2023 | Relationship between exposure and outcome not directly addressed |
| Maxwell 2020 | Population other than described in PICO question |
| Maxwell 2022 | Do not describe the desire exposure and outcome |
| Melendez-Torres 2014 | Inappropriate study design |
| Melendez-Torres 2020 | Inappropriate study design |
| Moran 2024 | Drug use other than chemsex |
| Nazli 2022 | Relationship between exposure and outcome not directly addressed |
| Nozza 2022 | Do not describe the desire exposure and outcome |
| O'Halloran 2019 | Do not describe the desire exposure and outcome |
| O'Halloran 2021 | Do not describe the desire exposure and outcome |
| Oldenburg 2016 | Drug use other than chemsex |
| Pan 2023 | Incomplete data |
| Pedrosa 2024 | Relationship between exposure and outcome not directly addressed |
| Peyriere 2023 | Do not describe the desire exposure and outcome |
| Pillet 2019 | Relationship between exposure and outcome not directly addressed |
| Plotzker 2017 | Drug use other than chemsex |
| Ramos 2025 | Population other than described in PICO question |
| Reyniers 2021 | Relationship between exposure and outcome not directly addressed |
| Ringshall 2022 | Do not describe the desire exposure and outcome |
| Rotsaert 2022 | Do not describe the desire exposure and outcome |
| Roux 2018 | Do not describe the desire exposure and outcome |
| Russ 2023 | Relationship between exposure and outcome not directly addressed |
| Saxton 2019 | Do not describe the desire exposure and outcome |
| Schecke 2019 | Incomplete data |
| Sewell 2018 | Incomplete data |
| Sewell 2019 | Do not describe the desire exposure and outcome |
| She 2025 | Incomplete data |
| Shover 2018 | Population other than described in PICO question |
| Sietins 2025 | Incomplete data |
| Sousa 2020 | Incomplete data |
| Sriperambudoori 2025 | Population other than described in PICO question |
| Storholm 2017 | Drug use other than chemsex |
| Strong 2023 | Inappropriate study design |
| Suzan-Monti 2018 | Relationship between exposure and outcome not directly addressed |
| *The Reflexive Logics*…2019* | Inappropriate study design |
| *The Use of*… 2021* | Population other than described in PICO question |
| Urban 2025 | Population other than described in PICO question |
| Vanbaelen 2023 | Do not describe the desire exposure and outcome |
| vandenElshout 2023 | Do not describe the desire exposure and outcome |
| vanWees 2024 | Inappropriate study design |
| Viamonte 2022 | Inappropriate study design |
| Vuylsteke 2021 | Do not describe the desire exposure and outcome |
| Wang 2020 | Relationship between exposure and outcome not directly addressed |
| Watson 2022 | Drug use other than chemsex |
| Wilson 2022 | Incomplete data |
| Winter 2022 | Do not describe the desire exposure and outcome |
| Xia 2020 | Do not describe the desire exposure and outcome |
| Zablotska 2013 | Incomplete data |
| Zapata 2025 | Drug use other than chemsex |
| Zhang 2022 | Relationship between exposure and outcome not directly addressed |
| Zucker 2022 | Relationship between exposure and outcome not directly addressed |

*For references without a listed author, the citation begins with the initial words of the title, formatted in italics

# **eFigure 1. Leave-one-out analysis of PrEP use prevalence by subgroups**

a

**
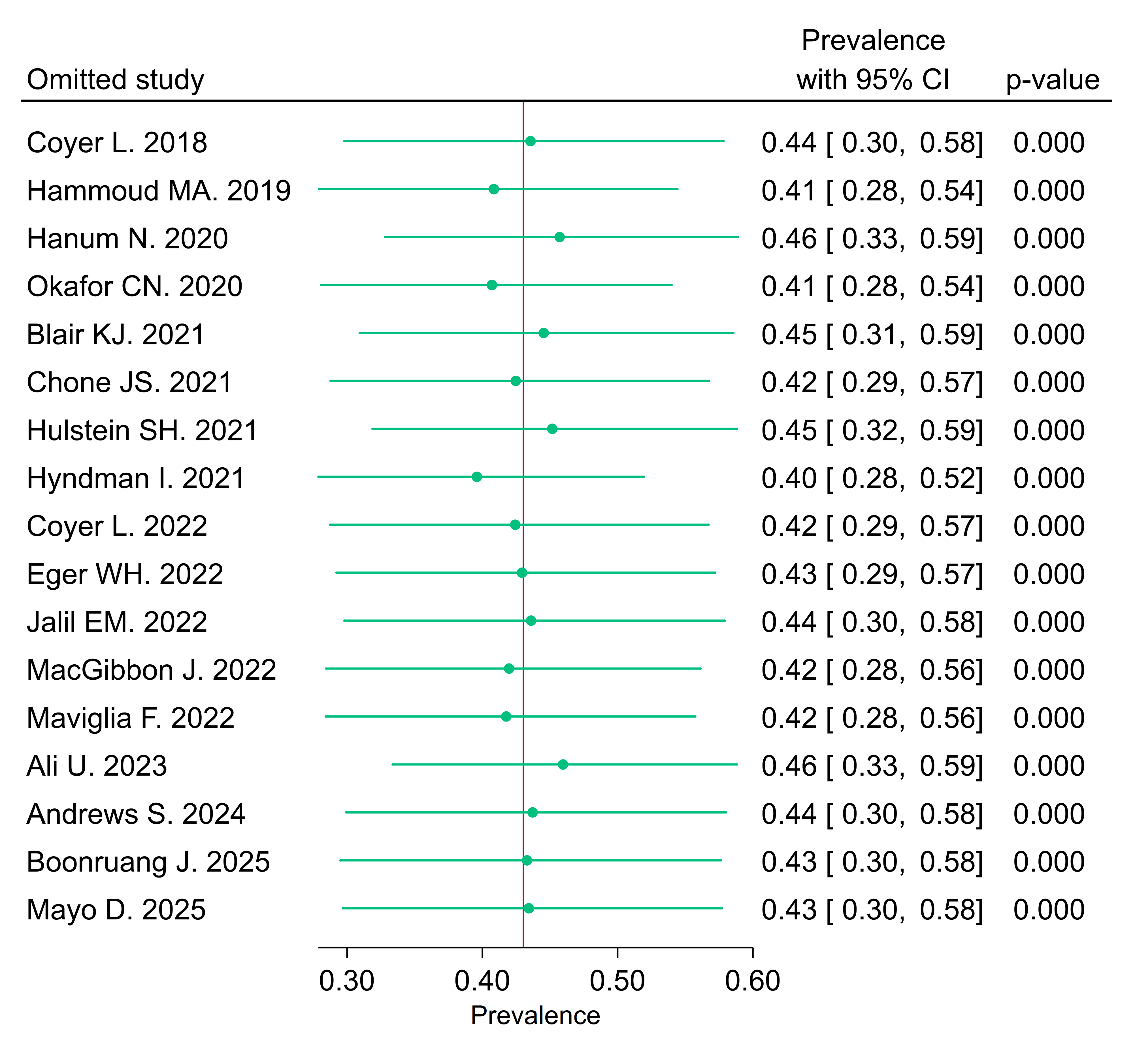
**

b

**
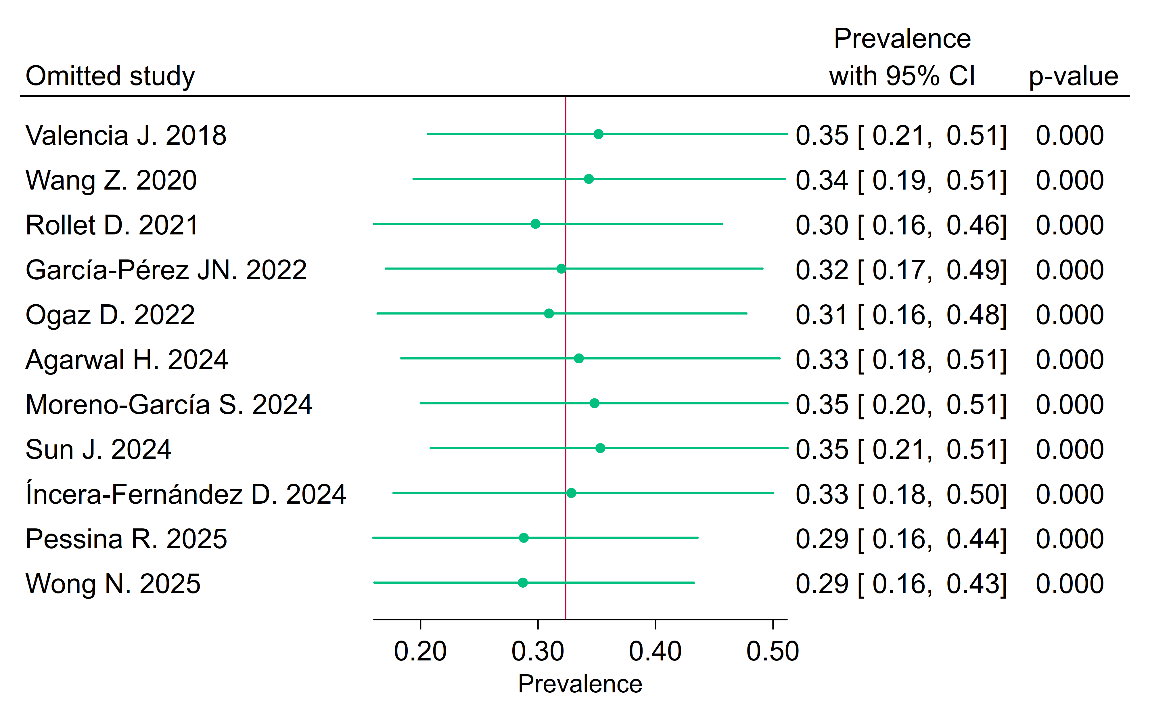
**

a: Leave-one-out analysis of PrEP use prevalence among adult cisgender GBMSM with *recent* chemsex engagement (less than 6 months).

b: Leave-one-out analysis of PrEP use prevalence among adult cisgender GBMSM *ever* chemsex engagement.

# **eTable 5. Meta-regression of PrEP Use Prevalence (recent chemsex engagement)**

|  | Coefficient | 95% Confidence Interval | p-value | I^2^ (%) | R^2^ (%) |
| --- | --- | --- | --- | --- | --- |
| Year | -0.057 | [-0.204, 0.090] | 0.446 | 98.60 | 0.00 |
| LMICs (vs. HICs) | -0.315 | [-0.869, 0.239] | 0.265 | 98.37 | 1.33 |
| Latin America (Asia and the Pacific) | -0.328 | [-1.224, 0.568] | 0.473 | 98.46 | 0.00 |
| Western and central Europe and North America (Asia and the Pacific) | 0.059 | [-0.537, 0.654] | 0.847 |  |  |

# **eTable 6. Meta-regression of PrEP Use Prevalence (ever chemsex engagement)**

|  | Coefficient | 95% Confidence Interval | p-value | I^2^ (%) | R^2^ (%) |
| --- | --- | --- | --- | --- | --- |
| Year | 0.086 | [-0.059, 0.231] | 0.234 | 97.24 | 1.36 |
| LMICs (vs. HICs) | -0.524 | [-1.312, 0.265] | 0.193 | 97.00 | 6.54 |
| Western and central Europe and North America (Asia and the Pacific) | 0.189 | [-0.489, 0.867] | 0.584 | 97.41 | 0.00 |

# **eTable 7. Meta-regression comparing recent vs. ever chemsex engagement: odds ratios**

|  | Coefficient | 95% Confidence Interval | p-value | I^2^ (%) | R^2^ (%) |
| --- | --- | --- | --- | --- | --- |
| Recent (vs. ever) | 0.605 | 0.383; 0.954 | 0.031 | 85.73 | 18.43 |

# **eFigure 2. Leave-one-out analysis of chemsex use by subgroups**

a

**
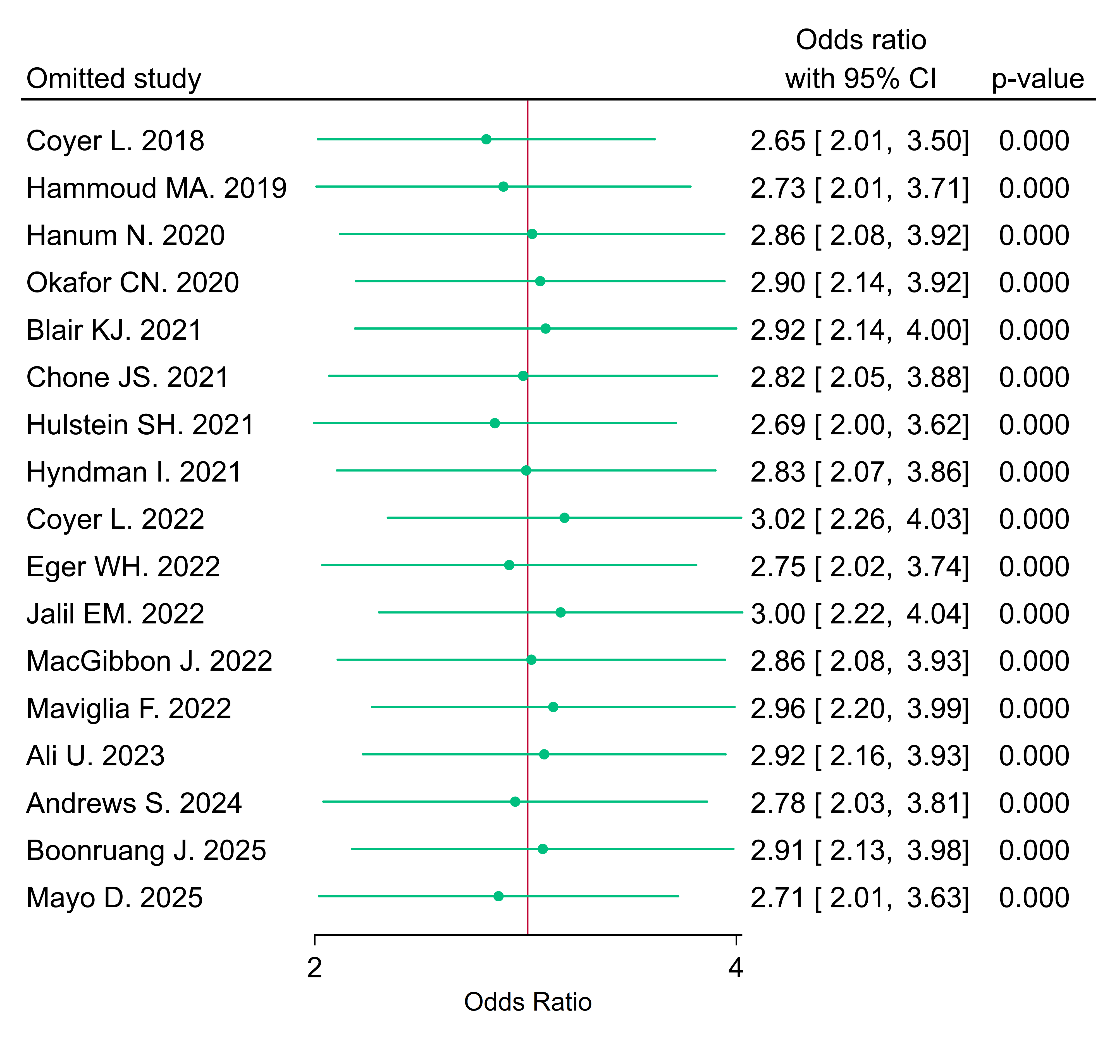
**

b

**
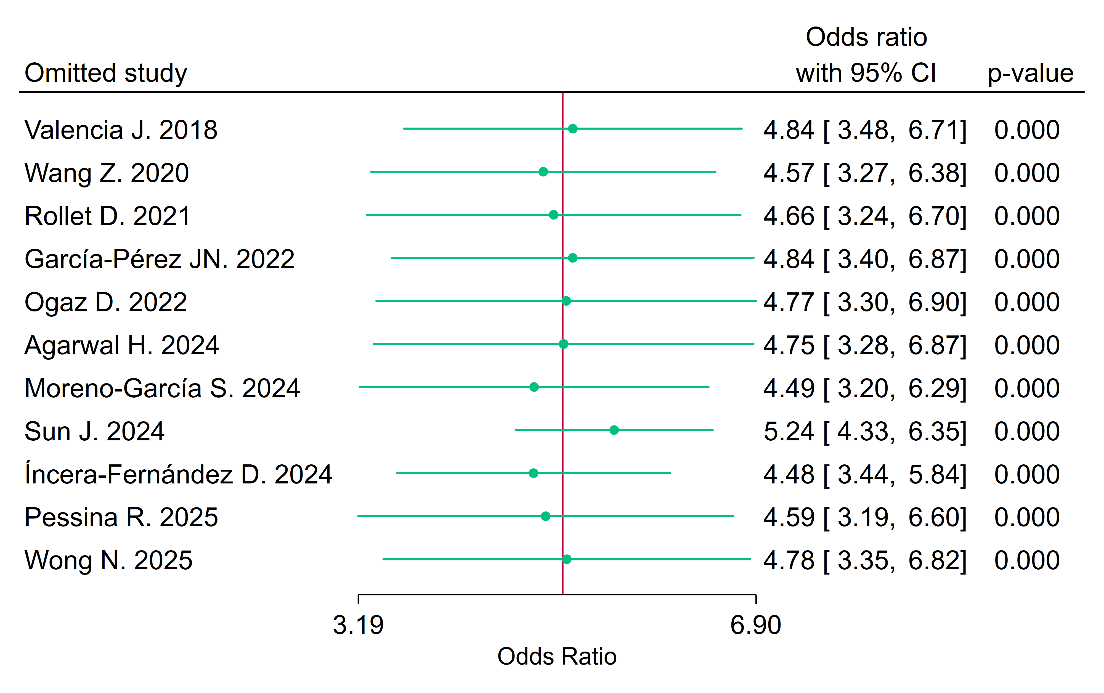
**

a: Leave-one-out analysis of the association between *recent* chemsex engagement and PrEP use among adult cisgender GBMSM.

b: Leave-one-out analysis of the association between *ever* chemsex engagement and PrEP use among adult cisgender GBMSM.

# **eTable 8. Meta-regression of the association between chemsex and PrEP use (recent chemsex engagement): odds ratios**

|  | Coefficient | 95% Confidence Interval | p-value | I^2^ (%) | R^2^ (%) |
| --- | --- | --- | --- | --- | --- |
| Year | 0.921 | [0.784, 1.081] | 0.314 | 88.87 | 1.57 |
| LMICs (vs. HICs) | 0.541 | [0.309, 0.944] | 0.031 | 84.60 | 27.47 |
| Latin America (Asia and the Pacific) | 0.550 | [0.231, 1.309] | 0.176 | 86.68 | 4.93 |
| Western and central Europe and North America (Asia and the Pacific) | 1.029 | [0.549, 1.931] | 0.928 |  |  |

# **eTable 9. Meta-regression of the association between chemsex and PrEP use (ever chemsex engagement): odds ratios**

|  | Coefficient | 95% Confidence Interval | p-value | I^2^ (%) | R^2^ (%) |
| --- | --- | --- | --- | --- | --- |
| Year | 1.013 | [0.850, 1.207] | 0.887 | 76.77 | 0.00 |
| LMICs (vs. HICs) | 0.460 | [0.214, 0.988] | 0.046 | 67.37 | 15.60 |
| Western and central Europe and North America (Asia and the Pacific) | 1.550 | [0.815, 2.945] | 0.181 | 70.31 | 5.82 |

# **eFigure 3. DOI plot assessing publication bias in the overall analysis**


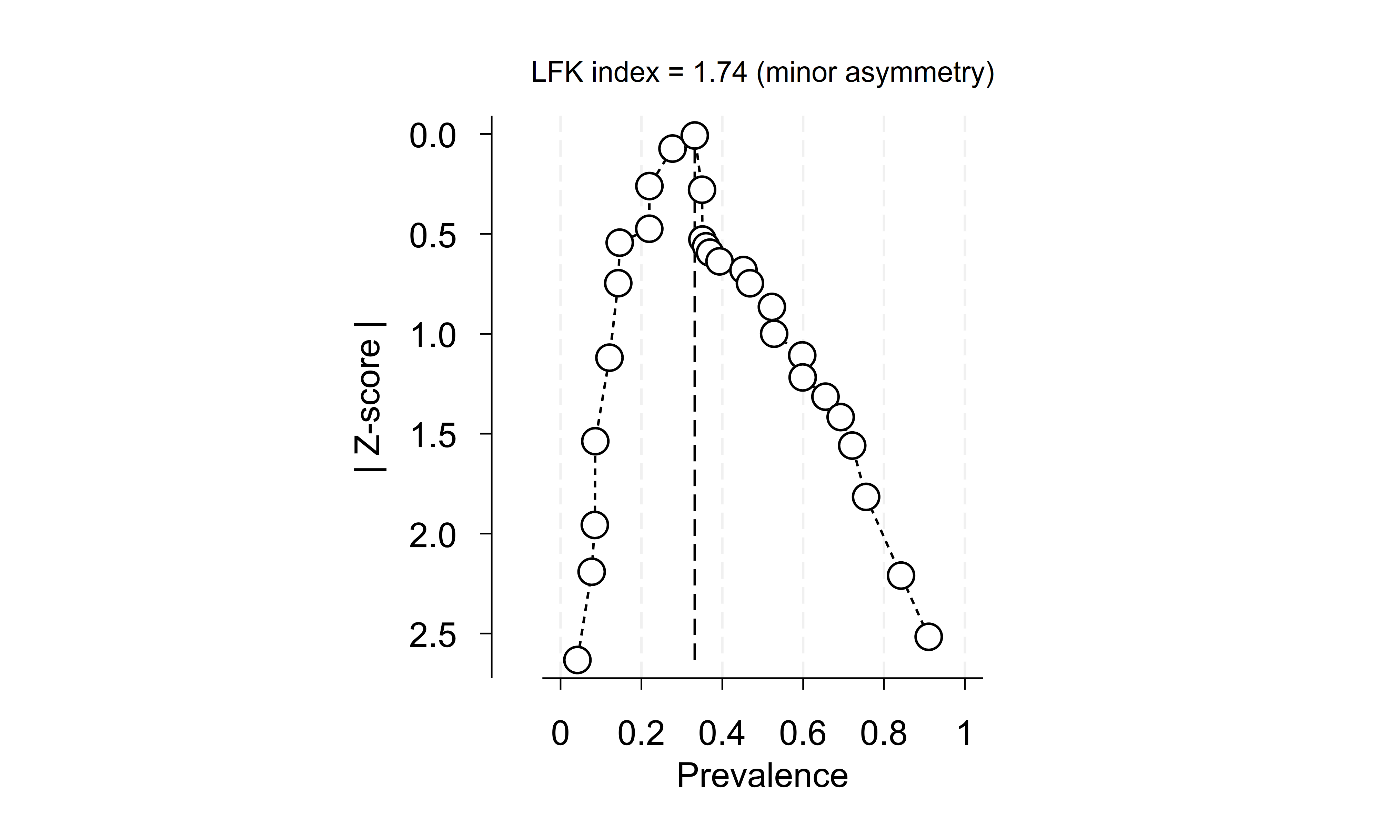


DOI plot and LFK index of asymmetry for PrEP use among adult cisgender MSM engaging in chemsex (*recent* or *ever* chemsex engagement).

# **eFigure 4. DOI plot assessing publication bias by subgroups**

a **
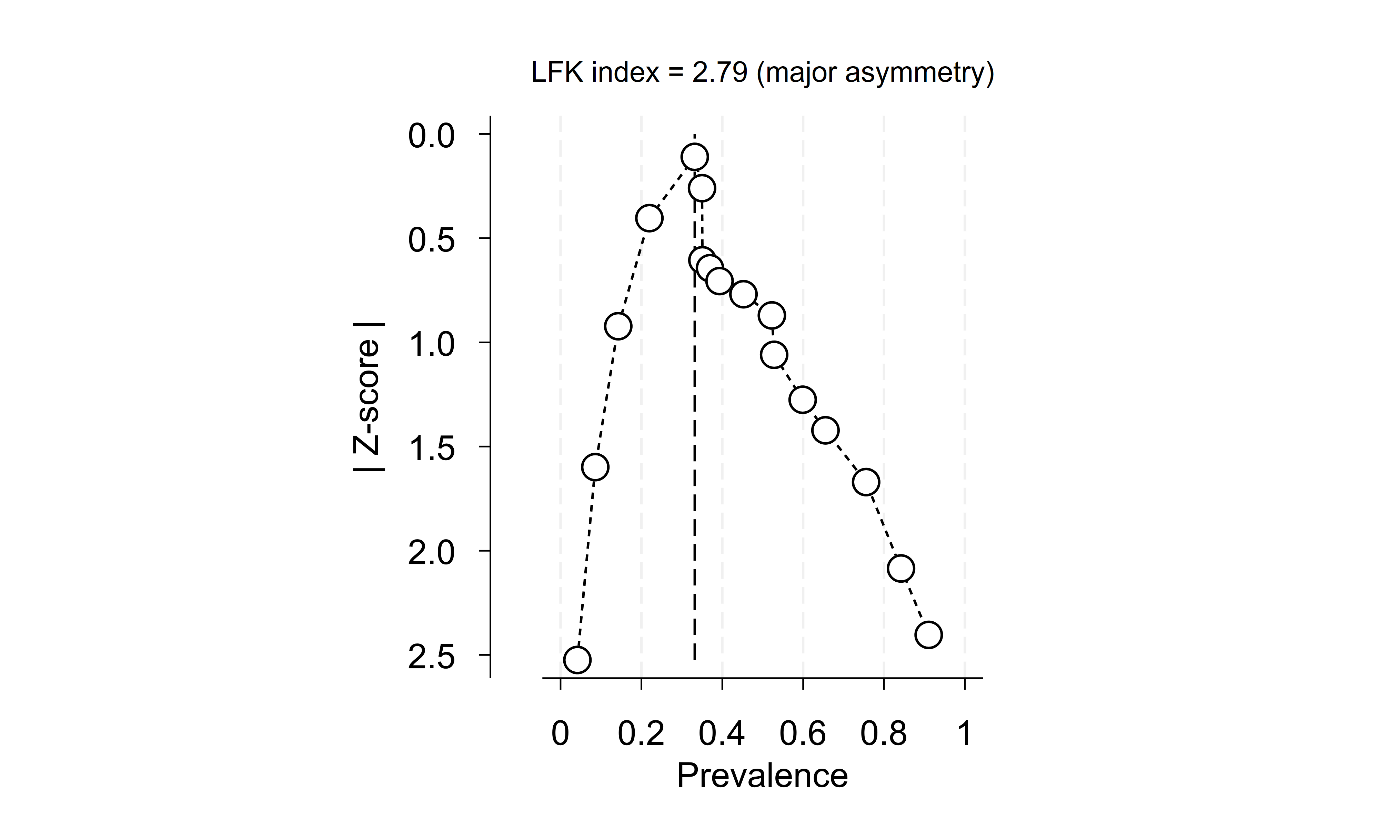
**

b

**
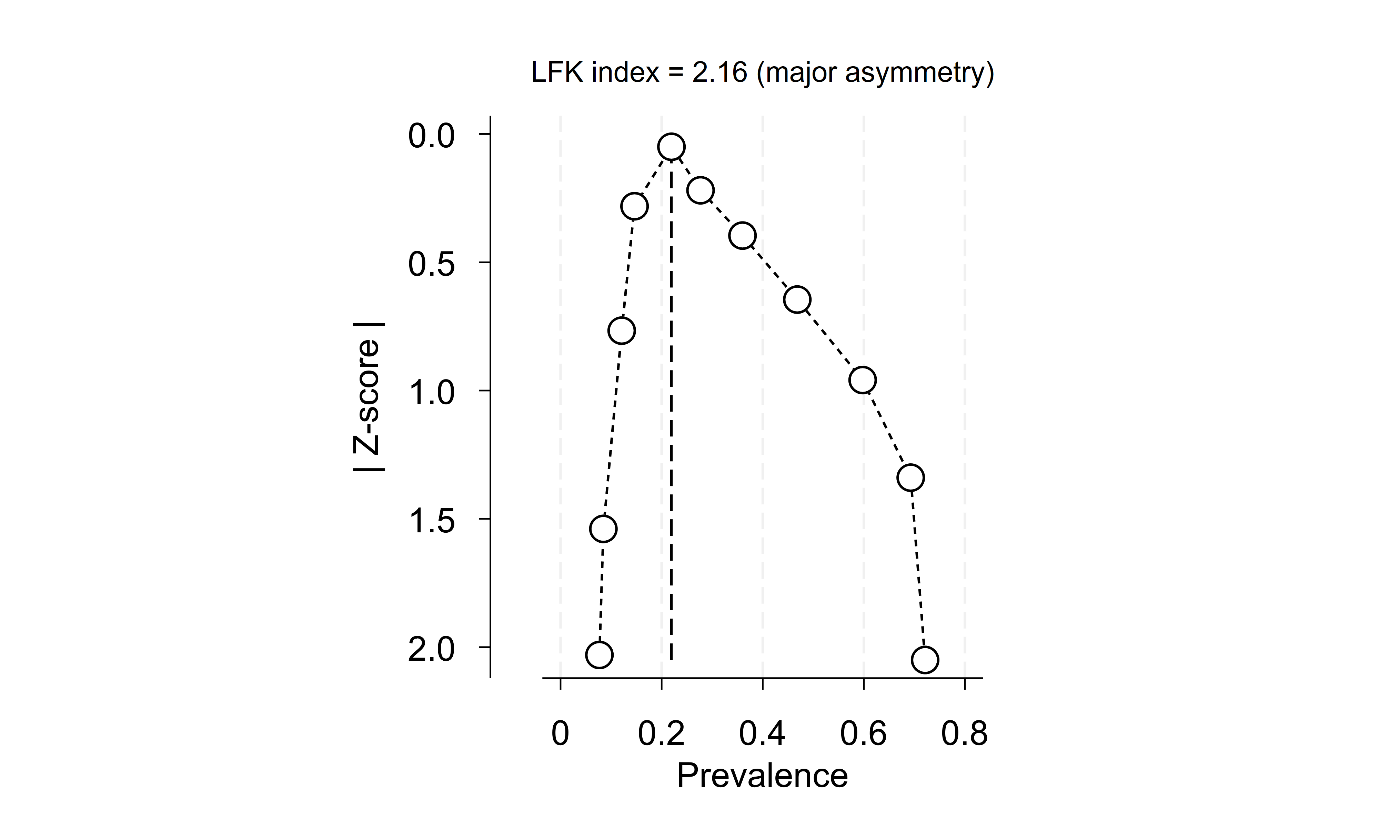
**

a: DOI plot and LFK index of asymmetry for PrEP use among adult cisgender GBMSM with *recent* chemsex engagement.

b: DOI plot and LFK index of asymmetry for PrEP use among adult cisgender GBMSM with *ever* chemsex engagement.

# **eFigure 5. Funnel plot assessing publication bias in the overall analysis**


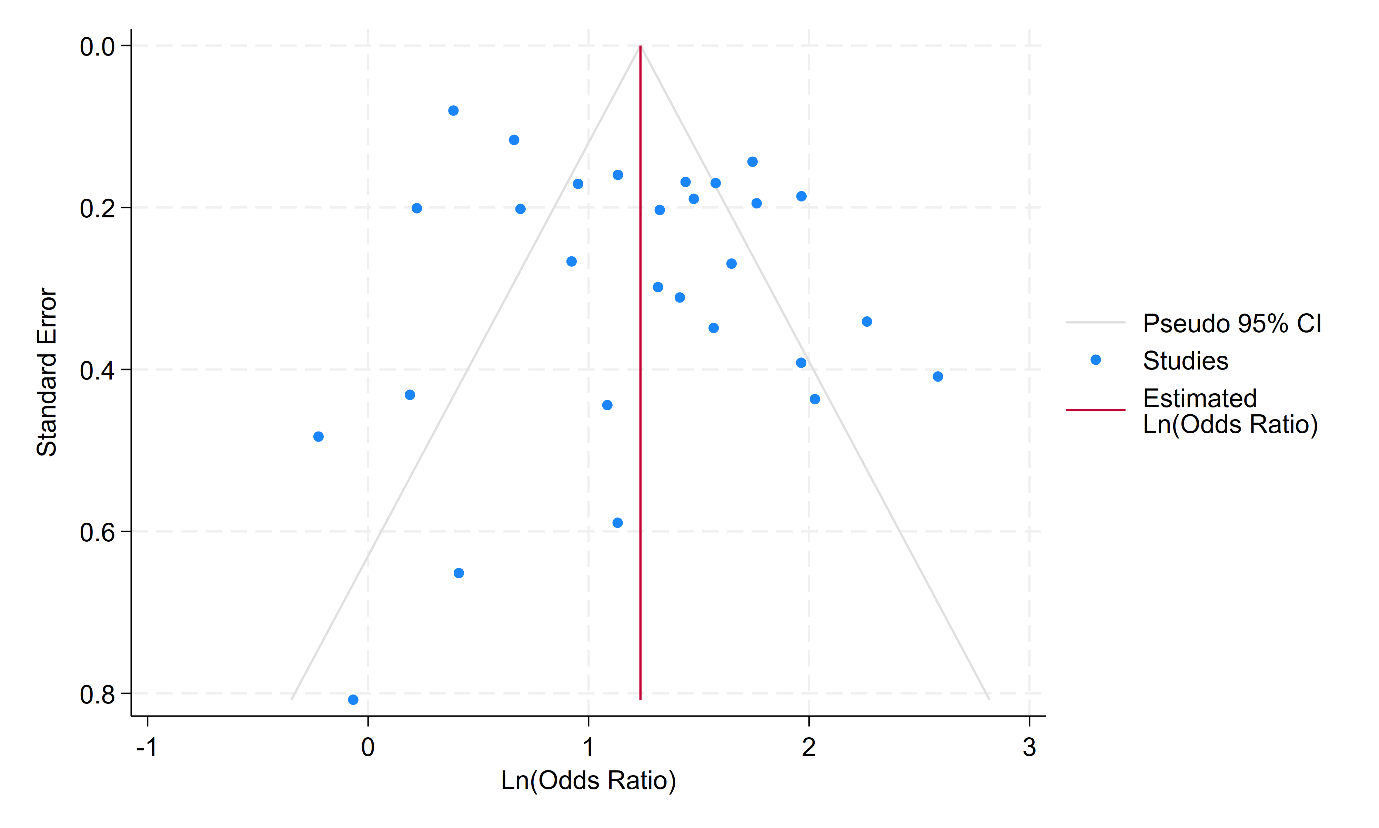


Publication bias for PrEP use among adult cisgender GBMSM engaging in chemsex (*recent* or *ever* chemsex engagement).

# **eFigure 6. Funnel plot assessing publication bias by subgroups**

a

**
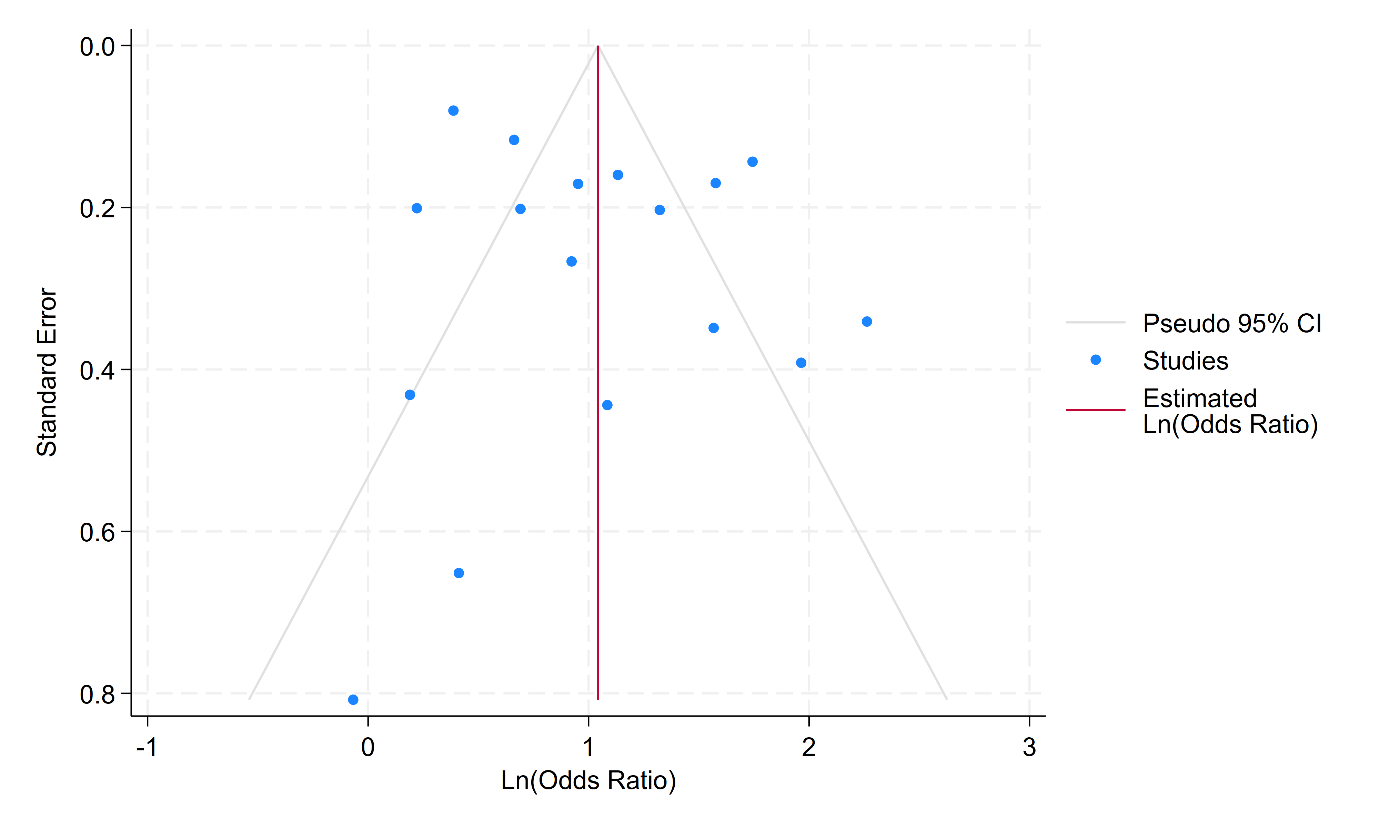
**

b

**
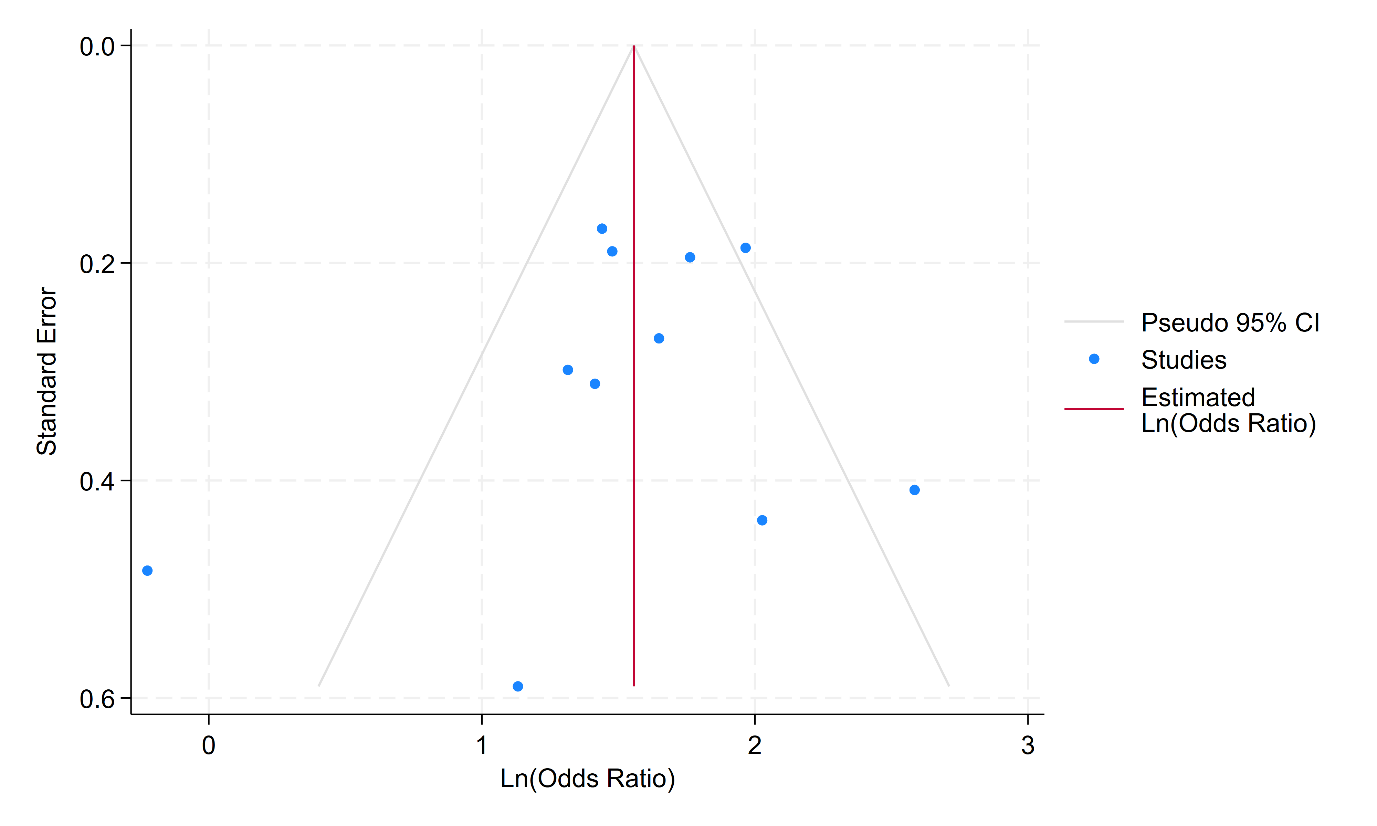
**

a: Publication bias for PrEP use among adult cisgender GBMSM with *recent* chemsex engagement.

b: Publication bias for PrEP use among adult cisgender GBMSM with *ever* chemsex engagement.
